# Identification, characterization, and transcription analysis of xylogen-like arabinogalactan proteins in rice (*Oryza sativa* L.)

**DOI:** 10.1186/s12870-014-0299-y

**Published:** 2014-11-18

**Authors:** Tengfei Ma, Haoli Ma, Heming Zhao, Huandong Qi, Jie Zhao

**Affiliations:** State Key Laboratory of Hybrid Rice, College of Life Sciences, Wuhan University, Wuhan, 430072 China

**Keywords:** Rice, XYLPs, Arabinogalactan protein, Expression analysis, Non-specific lipid transfer protein

## Abstract

**Background:**

Xylogen, a chimeric arabinogalactan protein containing a non-specific lipid transfer protein domain, can promote xylem cell differentiation. No comprehensive study has been carried out on the *XYLP* gene family in rice. As a first step in research on this gene family and as a useful strategy in general, a genome-wide analysis of the *OsXYLP* gene family is thus needed.

**Results:**

In this study, we identified 21 *XYLP* genes from the rice genome and comprehensively analyzed their protein structures, phylogenetic relationships, chromosomal locations, and gene duplication status. Our results indicate that gene duplication has played major roles in the expansion of the *OsXYLP* gene family. We used expressed sequence tag, microarray, massively parallel signature sequencing, and quantitative real-time PCR data to analyze *OsXYLP* gene expression during various developmental stages and under abiotic stress conditions. We found that many *OsXYLP* genes are abundantly expressed in vascular tissues and seeds, with some genes regulated under hormonal or abiotic stresses. In addition, we identified knockout mutants of *OsXYLP7* and *OsXYLP16* and discovered that the mutant *xylp7* has a defect in stem height.

**Conclusions:**

We analyzed expression profiles of 21 *XYLP* genes and characterized the structures and evolutionary relationships of their proteins. Our results demonstrate that the rice *XYLP* gene family may play roles in plant vascular system development and hormone signaling. Among the 21 detected *OsXYLPs*, 19 are newly identified genes encoding arabinogalactan proteins. Our results provide comprehensive insights that will assist future research on the biological functions of the rice *XYLP* gene family.

**Electronic supplementary material:**

The online version of this article (doi:10.1186/s12870-014-0299-y) contains supplementary material, which is available to authorized users.

## Background

Arabinogalactan proteins (AGPs) are a class of extracellular glycoproteins consisting of a core protein backbone and diverse type-II arabinogalactan (AG) polysaccharide chains made up of galactan and arabinose [1-4]. Typical AGP molecular weights range from 60 to 300 kDa. The protein backbones are usually rich in proline/hydroxyproline, alanine, serine, and threonine (PAST), with the hydroxyproline O-glycosylated by peripheral AG side chains that determine macromolecular heterogeneity [3,5]. AGPs are classified into several subclasses based on their core protein structures: classical AGPs, Lys-rich AGPs, AG peptides, non-classical AGPs, and chimeric AGPs [6-9]. According to their domain constitutions, chimeric AGPs can be further divided into three subclasses: fasciclin-like AGPs (FLAs) [7,10], xylogen-like proteins (XYLPs) [11,12], and phytocyanin-like AGPs (PLAs) [10,13,14]. Previous researchers have identified 98 AGPs in rice, including 11 classical AGPs, 15 AG peptides, 2 Lys-rich AGPs, 27 FLAs, 38 phytocyanin-like AGPs, and 3 non-classical AGPs [14-16]. AGPs can selectively bind to a synthetic dye, β-glucosyl Yariv reagent (β-GlcY). Although the precise underlying mechanism is unclear, this binding requires the presence of both the protein and AG chains. β-GlcY binding ability can thus be used as a distinguishing standard to identify AGPs [17,18]. Many studies on the biological function of AGPs have been performed using β-GlcY and polyclonal antibodies such as JIM8, JIM13, JIM14, LM2, and CCRC-M7 [19]. AGPs have been reported to be involved in various plant growth and developmental processes, such as cell expansion [20-22], cell proliferation [23-25], programmed cell death [26,27], cell wall plasticization [28], hormone response [29], salt tolerance [28,30], xylem differentiation [11], root growth and development [31], female and male gametogenesis [32-36], pollen tube growth [37,38], and zygotic division and embryo development [33,39-42].

Plant non-specific lipid-transfer proteins (nsLTPs), which are abundant small basic proteins that can transfer phospholipids between membranes, were first isolated from spinach leaves as phospholipid-binding proteins [43,44]. The lipid-binding properties of nsLTPs are derived from a unique structure: a region of eight strictly conserved cysteine residues. The eight cysteines bind to one another to form four disulfide bridges that give rise to a three-dimensional structure containing an internal hydrophobic cavityable to firmly bind lipids [44]. Xylogen, a 25–300-kDa glycoprotein, mediates local intercellular communication and is essential for tracheary element (TE) differentiation in *in vitro Zinnia elegans* xylogenic culture [44,45]. Xylogen is secreted from differentiating vascular cells and promotes the transformation of adjacent undifferentiated cells into TEs; it has a unique structure including AGP domains and an nsLTP domain, as typical structure of chimeric AGPs [11]. In a previous bioinformatic analysis of xylogen-type proteins in *Arabidopsis* [12], 13 *AtXYLP* (xylogen-like protein) genes with significant similarity to *ZeXYP1* were identified and their expression profiles were analyzed.

Genome-wide analysis is a useful strategy for the elucidation of biological functions of the *XYLP* gene family. In this study, we identified 21 *XYLP* genes in the rice (*Oryza sativa* L.) genome and conducted a phylogenetic analysis. To obtain further information about *OsXYLP* gene expression patterns, we evaluated publicly available resources such as microarray and massively parallel signature sequencing (MPSS) databases. We then validated the digital expression data obtained for these genes through quantitative real-time PCR (qRT-PCR). In addition, we identified the knockout mutants of *OsXYLP7* and *OsXYLP16* and found that *OsXYLP7* is involved in stem development. Our results provide a comprehensive understanding of *OsXYLPs* and may serve as a guide for research on the *OsXYLP* gene family.

## Results

### Identification of putative OsXYLPs

To identify xylogen-like proteins (XYLPs) in rice, we performed BLASTP searches across several rice protein databases using ZeXYP1, AtXYP1, and AtXYP2 protein sequences as queries [11]. After confirming the presence of nsLTP-like domains, AGP-like regions, and AG-type glycomodules and removing redundant sequences, we identified 21 OsXYLPs in rice (Table 1). To ensure the detection of all proteins in this family, we conducted additional BLASTP searches using protein sequences of the 21 identified OsXYLPs; these searches yielded no more XYLPs. Among the 21 OsXYLPs, we identified 19 new AGPs. The remaining 2 identified OsXYLPs, OsLTPL1 (OsLLA1) [16,46] and OsXYLP9 (OsLLA6) [46], were among 98 AGPs previously identified [14-16,46]. OsLTPL1 was first isolated as a β-GlcY-reactive arabinogalactan protein; and then OsLTPL1 and OsXYLP9 were identified as nsLTP-like AGPs.

**Table 1:** **The general information of rice** ***XYLP*** **genes**

**Gene** ^**a**^	**Subfamily** ^**b**^	**RGAP locus** ^**c**^	**RAP-DB locus** ^**d**^	**Chromosome location** ^**e**^	**Size(aa)** ^**f**^	**Signal** ^**g**^	**GPI** ^**h**^	**FL-cDNA** ^**i**^	**EST** ^**j**^	**MI** ^**k**^	**MPSS** ^**l**^
*OsLTPL1*	Clade A	LOC_Os03g26820	Os03g0385400	chr03:15307624..15309776	178	√	√	√	√	√	√
*OsXYLP2*	Clade A	LOC_Os03g26800	Os03g0385100	chr03:15300737..15301156	139	√	-	-	-	-	√
*OsXYLP3*	Clade A	LOC_Os07g30590	Os07g0489000	chr07:18102558..18103713	170	√	√	√	√	√	√
*OsXYLP4*	Clade A	LOC_Os07g43290	Os07g0625800	chr07:25920699..25922871	177	√	√	√	√	√	√
*OsXYLP5*	Clade A	LOC_Os03g09230	Os03g0192600	chr03:4820699..4822659	214	√	√	√	√	√	√
*OsXYLP6*	Clade B	LOC_Os03g20760	Os03g0323900	chr03:11751959..11753399	199	√	√	√	√	√	-
*OsXYLP7*	Clade B	LOC_Os05g41030	Os05g0489200	chr05:24045555..24047844	210	√	√	√	√	√	√
*OsXYLP8*	Clade B	LOC_Os01g59870	Os01g0814100	chr01:34626255..34628601	187	√	√	√	√	√	√
*OsXYLP9*	Clade C	LOC_Os07g07790	Os07g0174400	chr07:3910661..3911056	188	√	√	-	√	√	√
*OsXYLP10*	Clade C	LOC_Os07g07860	Os07g0174900	chr07:3947968..3949371	171	√	√	-	√	√	√
*OsXYLP11*	Clade C	LOC_Os03g57990	Os03g0794000	chr03:33028009..33029105	189	√	√	-	√	√	√
*OsXYLP12*	Clade C	LOC_Os07g07870	Os07g0175000	chr07:3950662..3951180	181	√	√	-	-	√	√
*OsXYLP13*	Clade C	LOC_Os03g57970	Os03g0793800	chr03:33023016..33023942	177	√	√	√	√	√	√
*OsXYLP14*	Clade C	LOC_Os07g07930	Os07g0175600	chr07:3980211..3981196	170	√	√	√	√	√	√
*OsXYLP15*	Clade C	LOC_Os04g38840	Os04g0462200	chr04:23093153..2309406	200	√	√	√	√	√	-
*OsXYLP16*	Clade D	LOC_Os07g09970	Os07g0198300	chr07:5313828..5318057	207	√	√	√	√	√	√
*OsXYLP17*	Clade D	LOC_Os03g58940	Os03g0804200	chr03:33563702..33566613	195	√	√	√	√	√	√
*OsXYLP18*	Clade D	LOC_Os03g07100	Os03g0167000	chr03:3626392..3628578	187	√	√	√	√	√	√
*OsXYLP19*	Clade D	LOC_Os06g47200	Os06g0686400	chr06:28617447..28620310	150	√	√	√	√	√	√
*OsXYLP20*	Clade D	LOC_Os03g46150	Os03g0664400	chr03:26096042..26096992	243	√	√	√	√	√	√
*OsXYLP21*	Clade D	LOC_Os08g42040	Os08g0532800	chr08:26554159..26557021	179	√	√	√	√	√	√

^a^Systematic designation given to rice *XYLPs*.

^b^OsXYLPs are divided into four clades according to the sequence homology of their protein backbones.

^c^and ^d^Locus numbers assigned by RGAP (Rice Genome Annotation Project, http://rice.plantbiology.msu.edu/) and RAP-DB (Rice Annotation Project Database, http://rapdb.dna.affrc.go.jp/), which can be converted by ID converter (http://rapdb.dna.affrc.go.jp/tools/converter/).

^e^Chromosomal localization of rice *XYLP* genes.

^f^Length of the open reading frame in amino acids.

^g^N-terminal signal sequence predicted by SignalP 3.0 (http://www.cbs.dtu.dk/services/SignalP/).

^h^GPI anchor signal predicted by big-PI (http://mendel.imp.ac.at/gpi/plant_server.html).

^i^^~^^l^Full-length cDNA; Expressed sequence tag profiles; microarray data; massively parallel signature sequencing.

√, exist; −, not exist.

We performed a multiple sequence alignment on the nsLTP-like domains of 21 OsXYLPs and 13 AtXYLPs to clarify the sequence characteristics of OsXYLPs (Additional file 1: Figure S1). It is noteworthy that the distribution of eight cysteine (Cys) residues is highly conserved, following an C-X-C-X-CC-X-CXC-X-C-X-C pattern, in both OsXYLPs and AtXYLPs. The hydrophobicity of the residue between Cys5 (C5) and Cys6 (C6) is also conserved, with the residue always leucine, isoleucine, or valine (Additional file 1: Figure S1). The conserved nature of the eight Cys residues and the hydrophobic residue, which in combination are involved in the formation of the three-dimensional structure that can firmly bind lipids, implies their important contribution to lipid-binding ability.

### Protein structure and phylogenetic analysis

**Figure 1:**
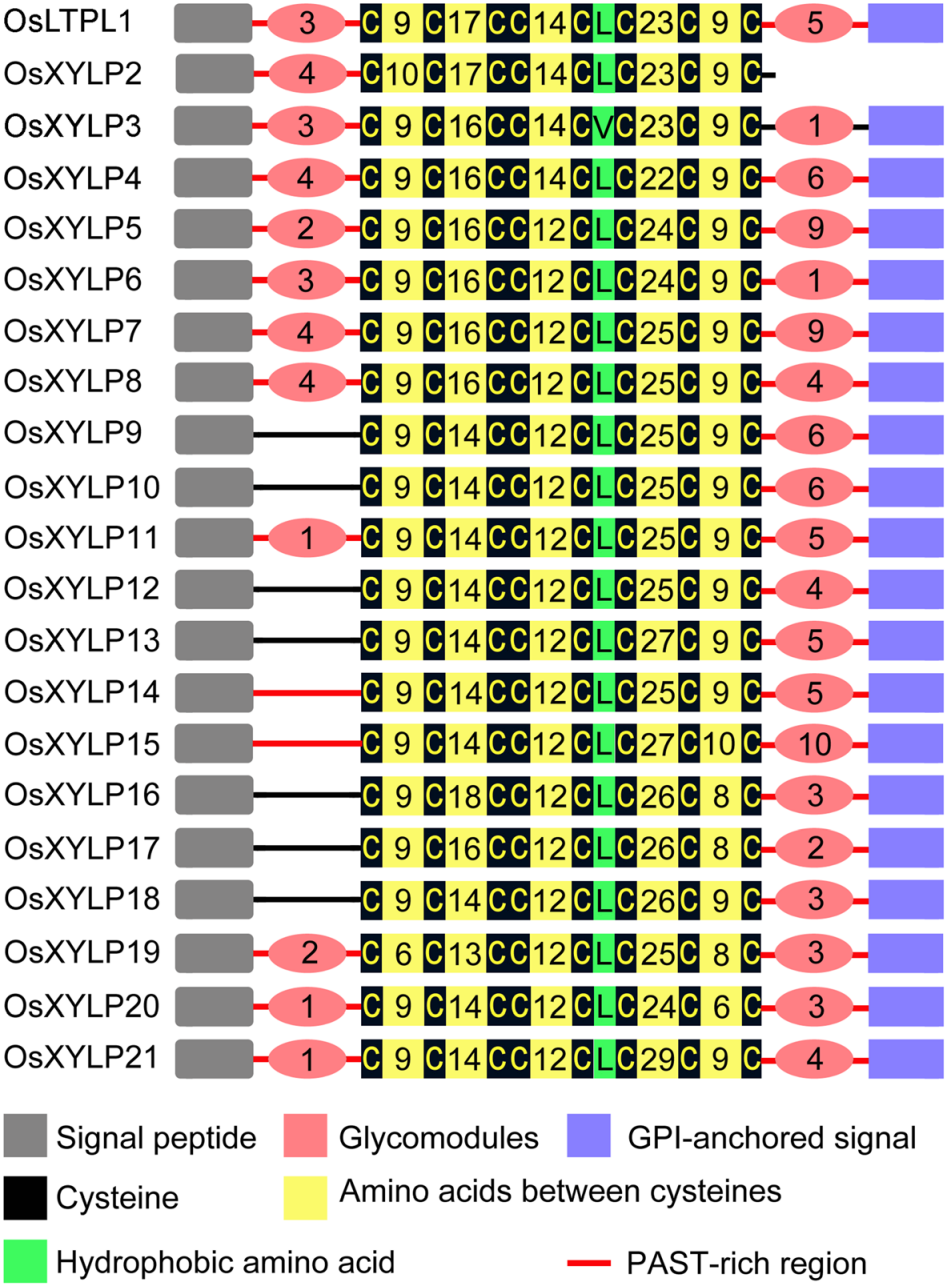
**Protein structure of rice XYLPs.** Gray boxes indicate the secretory signal sequence predicted by SignalP. The violet boxes indicate predicted the GPI-anchored signal. Dark red straights indicate glycoprotein-like Pro/Ala/Ser/Thr-rich regions (PAST > 35%). Light red circles with number indicate putative AG glycomodules and its number. Yellow and black boxes indicate nsLTP domains; black boxes indicate the eight conserved cysteine residues; the numbers in yellow boxes means the number of amino acid residues; the green boxes show the hydrophobic residues between C5 and C6.

The OsXYLP protein sequences were submitted to several bioinformatic websites to predict the presence of signal peptides, glycosylphosphatidylinositol (GPI)-anchored signals, N-glycosylation sites, and AG glycomodules (Additional file 2: Table S1). All 21 OsXYLPs were expected to have an N-terminal signal peptide for targeting to the endoplasmic reticulum. All OsXYLPs except for OsXYLP2 were found to be GPI anchor proteins, indicating that these proteins might localize in the plasma membrane (Figure 1). In addition, putative AG glycomodules in all OsXYLPs were found to be distributed in the PAST-rich region before and/or after the nsLTP-like domain (Figure 1). Moreover, N-glycosylation sites in most of the OsXYLPs were located in the nsLTP-like domain and the PAST-rich region (Additional file 2: Table S1). The existence of signal peptides and AG glycomodules suggest that the 21 OsXYLPs may be chimeric AGPs.

**Figure 2:**
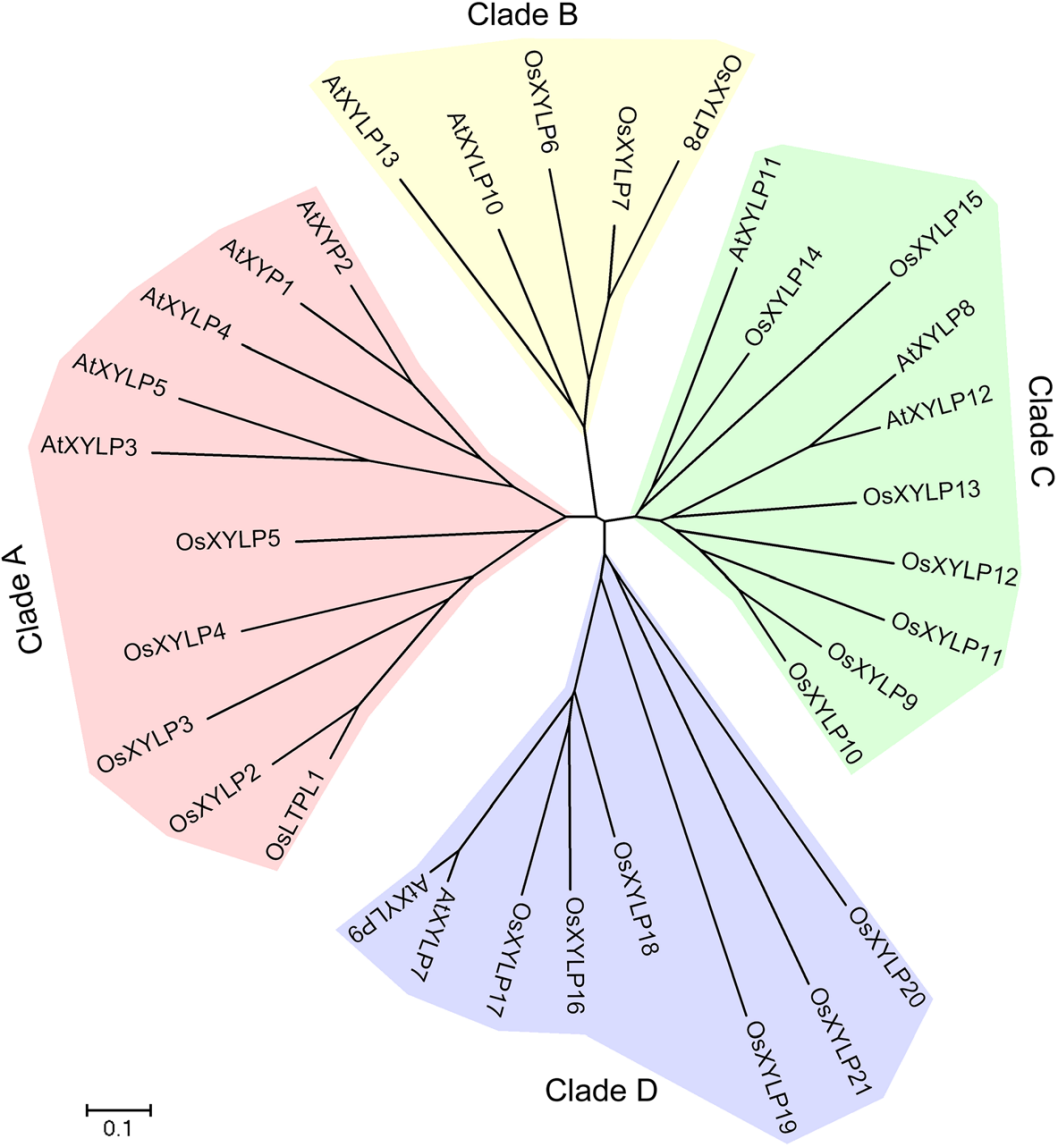
**Phylogenetic relationship of XYLPs between rice and** ***Arabidopsis*** **.** Four clades of XYLPs are show on different color backgrounds. Scale bar represent 0.1 amino acid substitution per site.

Using the aligned full-length OsXYLP and AtXYLP protein sequences, we obtained an unrooted phylogenetic tree showing their phylogenetic relationships (Figure 2). With a few exceptions, all XYLPs in the tree are clustered according to their protein sequence homologies into four distinct, strongly supported clades (A–D). Family members with high sequence homology therefore cluster together in the tree. For instance, five XYLPs each from rice and *Arabidopsis* are placed in Clade A, with cysteine residues distributed following the conserved pattern: C-X_9/10_-C-X_16/17_-CC-X_12/14_-C-L/V/I-C-X_22/23/24_-C-X_7/8/9_-C (Figures 1 and 2). Clade B consists of five XYLPs, three OsXYLPs, and two AtXYLPs (Figure 2). The distribution of the eight cysteine residues in the 10 XYLPs in Clade C displays a highly conserved pattern: C-X_9_-C-X_14_-CC-X_12_-C-L/V-C-X_25/27_-C-X_9/10_-C (Figures 1 and 2). In addition, the putative AG glycomodules in all 10 XYLPs are located between the nsLTP-like domain and the GPI anchor signal. The major difference between clades A, B, and C vs. clade D is that OsXYLP19, OsXYLP20, and OsXYLP21 in the latter have low similarity to other XYLPs. Representatives of rice and *Arabidopsis* are present in each clade in the phylogenetic tree. Within each clade, species-specific XYLPs from rice and *Arabidopsis* are grouped separately, indicating that the evolutionary expansions of XYLPs in rice and *Arabidopsis* have occurred independently.

### Chromosomal localization and gene duplication

**Figure 3:**
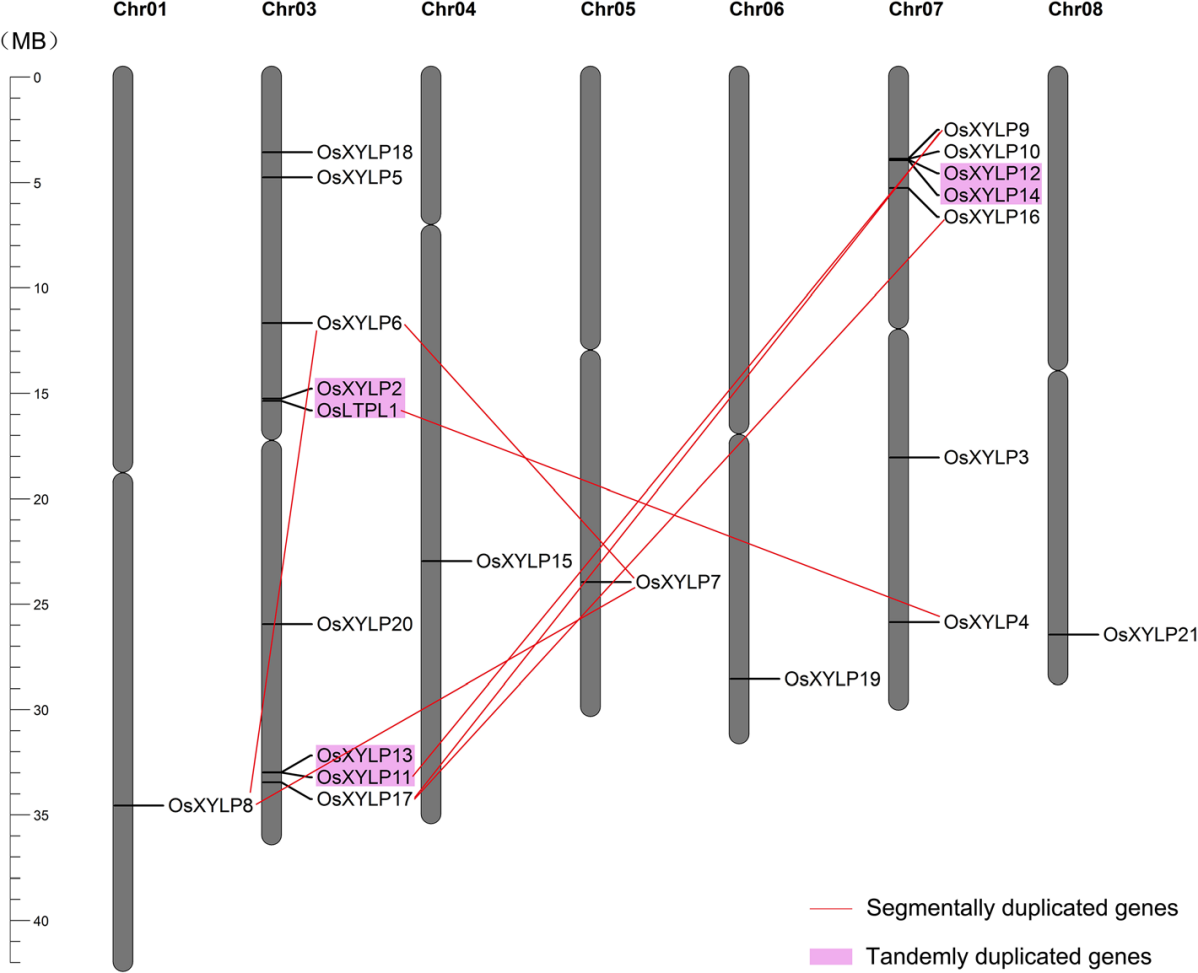
**Chromosomal localization and gene duplication events of** ***OsXYLP*** **genes.** Chromosome numbers are indicated at the top of each chromosome. The cleavages on the chromosomes indicate the position of centromeres. Genes present on duplicated segments of genome are connected by red lines, and tandem duplicated genes are marked with purple background.

We obtained the exact coordinates and orientations of *OsXYLP* genes from the Rice Genome Annotation Project (RGAP) database. The approximate locations of these genes are marked on the rice chromosome sketch shown in Figure 3. The *OsXYLP* genes are located on seven rice chromosomes: nine genes on chromosome 3, seven genes on chromosome 7, and one gene each on chromosomes 1, 4, 5, 6, and 8 (Figure 3). The *OsXYLPs* thus appear to be preferentially distributed.

We also investigated segmental and tandem duplications in the *OsXYLP* gene family. We found that nine *OsXYLP* genes (*OsLTPL1* and *OsXYLPs 4*, *6*, *7*, *8*, *9*, *11*, *16*, and *17*) located in the duplicated chromosomal segments of rice chromosomes mapped by RGAP with a maximal distance between collinear gene pairs of 500 kb (Figure 3). Additionally, six genes (*OsLTPL1* and *OsXYLPs 2, 11, 12, 13,* and *14*) are tandemly duplicated and separated by no more than five intervening genes. To summarize, 13 *OsXYLP* genes are associated with segmental and tandem duplications, indicating that evolution in this gene family has involved a large number of duplication events.

### Expression patterns of *OsXYLP* gene

Expression patterns are important for analyzing the function of target genes. To investigate expression patterns of *OsXYLP* genes, we accordingly investigated three publicly available resources: expressed sequence tag (EST) profiles, MPSS tags, and microarray data.

We examined the availability of EST and full-length cDNA data by searching the Rice Annotation Project Database locus of *OsXYLP* genes in the UniGene database at NCBI (http://www.ncbi.nlm.nih.gov/unigene/) (Table 1). We discovered that 19 of 21 *OsXYLP* genes are represented by at least one full-length cDNA or EST. Both full-length cDNAs and ESTs are reported for 16 genes, whereas 3 genes are only represented by an EST. The data indicate that the *OsXYLP* genes, except for *OsXYLP2*, are expressed (Table 1). The EST data demonstrate that four genes are tissue-specifically expressed: *OsLTPL1* in stems, *OsXYLP13* and *OsXYLP21* in shoot apical meristem (SAM), and *OsXYLP18* in panicles (Additional file 3: Table S2).

MPSS is a sensitive quantitative method for gene expression analysis [47]. To analyze the expression pattern of the 21 *OsXYLP* genes, we obtained two 17-base and 20-base signatures in 10 different organs and tissues of rice from the MPSS database. MPSS signatures for 16 *OsXYLP* genes were available in at least one of the two libraries (Additional file 4: Table S3). Differential expression abundances, represented by the number of tags (transcripts per million [tpm]), were classified to indicate low (<50 tpm), moderate (50–500 tpm), and strong (>500 tpm) expression. Eight and seven genes displayed strong and moderate expression levels, respectively, and four genes were expressed at a low level (Additional file 4: Table S3). It is noteworthy that 10 genes showed abundant or specific expression in roots, leaves, stems, and panicles. The results of this analysis are consistent with the predicted roles of *OsXYLP* genes in vascular system development.

**Figure 4:**
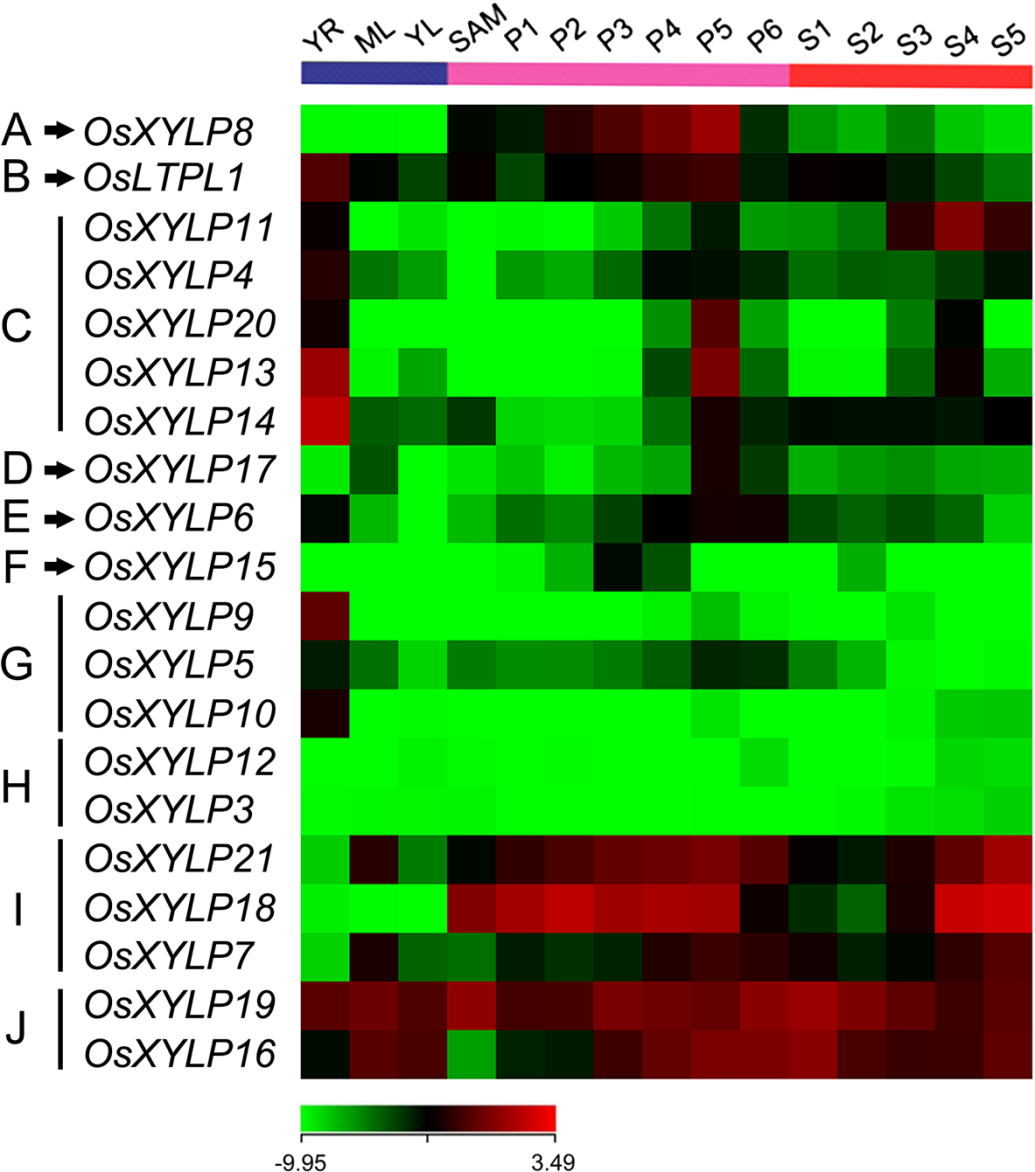
**Expression profiles of** ***OsXYLP*** **genes in various organs and tissues.** The microarray data (GSE6893) of *OsXYLP* genes expression are analyzed. A heat map representing hierarchical clustering of average log signal values of *OsXYLP* genes in various developmental stages are generated (samples are indicated at the top of each lane: YR, roots from 7-day-old seedlings; ML, mature leaves; YL, leaves from 7-day-old seedling, different stages of panicle development: SAM, up to 0.5 mm; P1, 0–3 cm; P2, 3–5 cm; P3, 5–10 cm; P4, 10–15 cm; P5, 15–22 cm; P6, 22–30 cm and different stages of seed development: S1, 0–2 dap (days after pollination); S2, 3–4 dap; S3, 5–10 dap; S4, 11–20 dap; S5, 21–29 dap). Genes are divided into 10 groups: **(A)** SAM, P1-P6, S1-S5; **(B)** all examined organs and tissues; **(C)** YR, P4-P6; **(D)** ML, P5, P6; **(E)** YR, P4-P6; **(F)** P3; **(G)** YR; **(H)** low expression in all examined organs and tissues; **(I)** SAM, P1-P6, S3-S5; **(J)** all examined organs and tissues. The color scale (representing average log signal values) is shown at the bottom.

Microarrays provide a high-throughput approach for the analysis of gene expression patterns. Microarray data were obtained from a previous study of *OsXYLP* gene expression in various tissues, including young roots (YR), mature leaves (ML), young leaves (YL), shoot apical meristem (SAM), and various stages of panicle (P1–P6) and seed (S1–S5) development [48]. A hierarchical cluster analysis was performed by using the logarithmic signal values of *OsXYLP* genes (Additional file 5: Table S4) revealed that 20 of the 21 *OsXYLPs* genes are expressed in at least one vegetative or reproductive developmental stage (Figure 4). *OsXYLP8* is abundantly expressed across the panicle development process (Figure 4A), while *OsLTPL1* is expressed in all examined organs and tissues (Figure 4B). Five genes (*OsXYLP4*, *OsXYLP11*, *OsXYLP13*, *OsXYLP14*, and *OsXYLP20*) are mainly expressed in YR and P5 (Figure 4C). High expression levels were indicated for *OsXYLP17* in P5 (Figure 4D), *OsXYLP6* in YR and P4–P6 (Figure 4E), and *OsXYLP15* in P3 (Figure 4F). *OsXYLP5*, *OsXYLP9*, and *OsXYLP10* are highly expressed in YR (Figure 4G). The expression levels of *OsXYLP3* and *OsXYLP12* are relatively low in all examined organs and tissues (Figure 4H). *OsXYLP7*, *OsXYLP18*, and *OsXYLP21* are highly expressed in panicles and seeds (Figure 4I), while the expression levels of *OsXYLP16* and *OsXYLP19* are high in all examined organs and tissues (Figure 4J).

**Figure 5:**
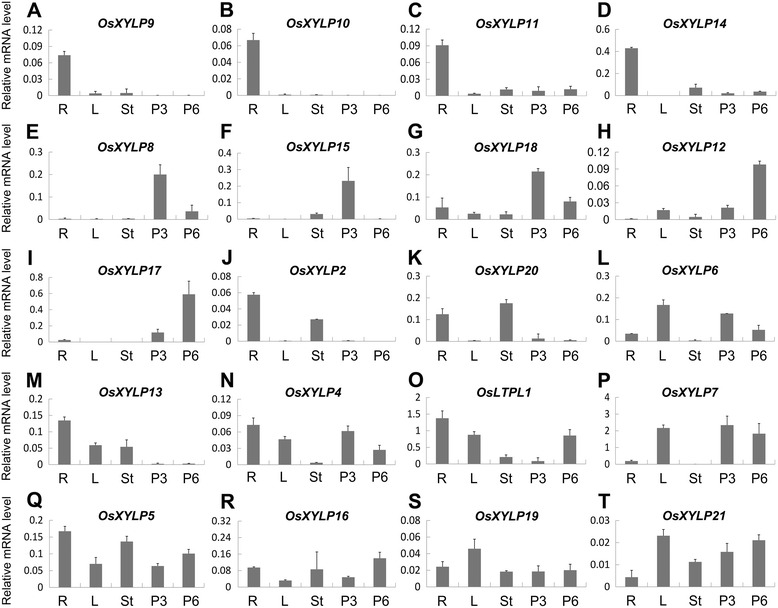
**Real-time PCR analysis of representative** ***OsXYLP*** **genes in different developmental stages of vegetative and reproductive tissues and organs.** The expression levels of *OsXYLP* genes in different tissues and organs **(A-T)**. R, 7-day-old roots; L, 7-day-old leaves; St, 60-day-old stems; P3, 5–10 cm panicles; P6, 22–30 cm panicles. Error bars indicate standard deviations of independent biological replicates (n =2 or more).

To validate the results of the digital expression analysis, we examined the expression levels of *OsXYLP* genes in five different tissues by qRT-PCR. The resulting gene expression patterns were in general agreement with the microarray and MPSS tag data (Figure 5). According to our PCR results, *OsXYLP9*, *OsXYLP10, OsXYLP11,* and *OsXYLP14* are especially expressed in roots (R) (Figure 5A–D), *OsLTPL8*, *OsXYLP15*, and *OsXYLP18* are predominantly expressed in P3 (Figure 5E–G), *OsXYLP12* and *OsXYLP17* are mainly expressed in P6 (Figure 5H and 5I), *OsXYLP2* and *OsXYLP20* are mainly expressed in roots and leaves (L) (Figure 5J and 5K), and *OsXYLP6* is mainly expressed in leaves and P3 (Figure 5L). Four genes are mostly expressed in three tissues: *OsXYLP13* in roots, leaves, and stems (Figure 5M), *OsXYLP4* in roots, leaves, and P3 (Figure 5N), *OsLTPL1* in roots, leaves, and P6 (Figure 5O), and *OsXYLP7* in leaves, P3, and P6 (Figure 5P). In contrast, no obviously specific expressions were observed for *OsXYLP5*, *OsXYLP16, OsXYLP19,* and *OsXYLP21* genes (Figure 5Q–T).

### Expression profiles of *OsXYLP* genes under abiotic stresses and hormone treatments

**Figure 6:**
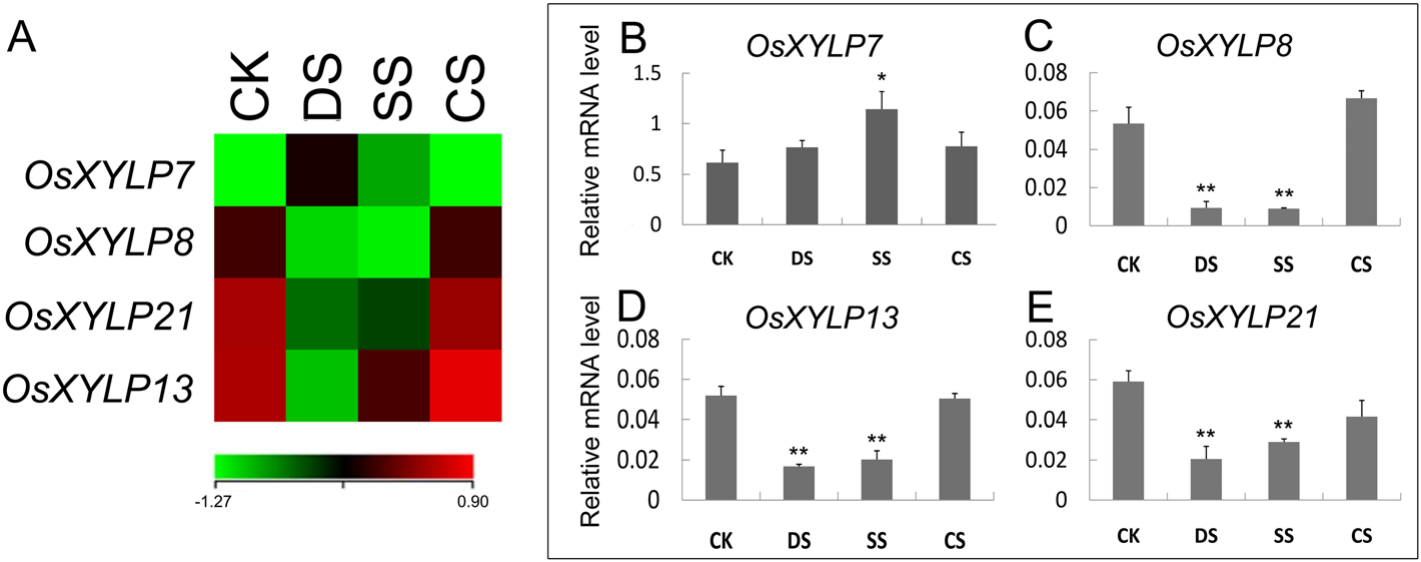
**Differential expression profiles of** ***OsXYLP*** **genes under abiotic stresses.** The microarray data (GSE6901) of gene expression under various abiotic stresses (CK, control; DS, drought stress; SS, salt stress; CS, cold stress) were used for cluster display. The average log signal values of *OsXYLP* genes are presented by a heat map. Under any of the given abiotic stress conditions, genes that exhibited ≥ 2-fold differential expression are shown **(A)**. Real-time PCR were performed on these genes **(B-E)**. The significance of difference between the controls and treatments are determined by using Origin 7.5, and are represented by two asterisks (**P < 0.01) and one asterisk (*0.01 < P < 0.05). The color scale (representing average log signal values) is shown at the bottom.

We analyzed the microarray data of 7-day-old seedlings under drought, salt, and cold stresses to investigate the abiotic stress response of *OsXYLPs*. Our results indicate that *OsXYLP7* expression is up-regulated by drought stress, whereas *OsXYLP8*, *OsXYLP13*, and *OsXYLP21* are down-regulated by drought and salt stresses (Figure 6). To verify the above results, we used qRT-PCR to detect the expression levels of these four genes in 7-day-old seedlings under three stress conditions for 3 hours (Figure 6B–E). The expression of *OsXYLP7* was up-regulated under salt stress (Figure 6B), while *OsXYLP8*, *OsXYLP13*, and *OsXYLP21* were significantly down-regulated by drought and salt stresses (Figure 6C–E). These results suggest that some *OsXYLP* genes may participate in abiotic stress pathways and play roles in the response to these stresses, especially drought and salt stresses.

**Figure 7:**
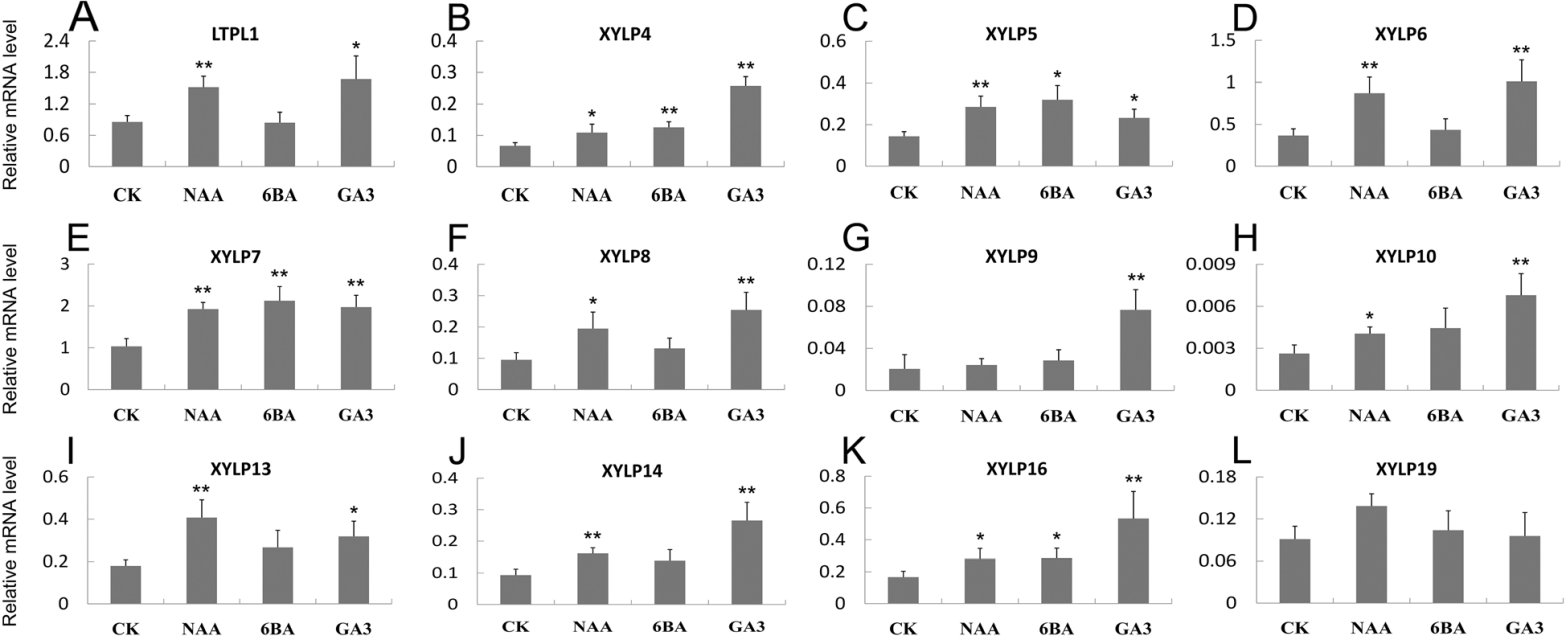
**Real-time PCR analysis of** ***OsXYLP*** **genes under NAA, 6-BA and GA treatments.** The expression levels of *OsXYLP* genes under different treatments **(A-L)**. The significance of difference between the controls and treatments are determined by using Origin 7.5, and are represented by two asterisks (**P < 0.01) and one asterisk (*0.01 < P < 0.05). CK, control.

We used qRT-PCR to examine transcriptional levels of 12 representative *OsXYLP* genes under NAA, 6-BA, and GA treatments (Figure 7). Except for *OsXYLP9* and *OsXYLP19*, the examined *OsXYLP* genes were up-regulated significantly in seedlings subject to NAA treatment (Figure 7). Only four genes (*OsXYLP4, OsXYLP5, OsXYLP7*, and *OsXYLP16*) displayed significant up-regulation under 6-BA treatment (Figure 7B,C,E, and K). Except for *OsXYLP19*, the expression levels of all examined genes were increased under GA treatment (Figure 7L). These results indicate that *OsXYLPs* may play roles in responses to these hormones.

### Comparative expression analysis of *OsXYLP* and *AtXYLP* genes

**Figure 8:**
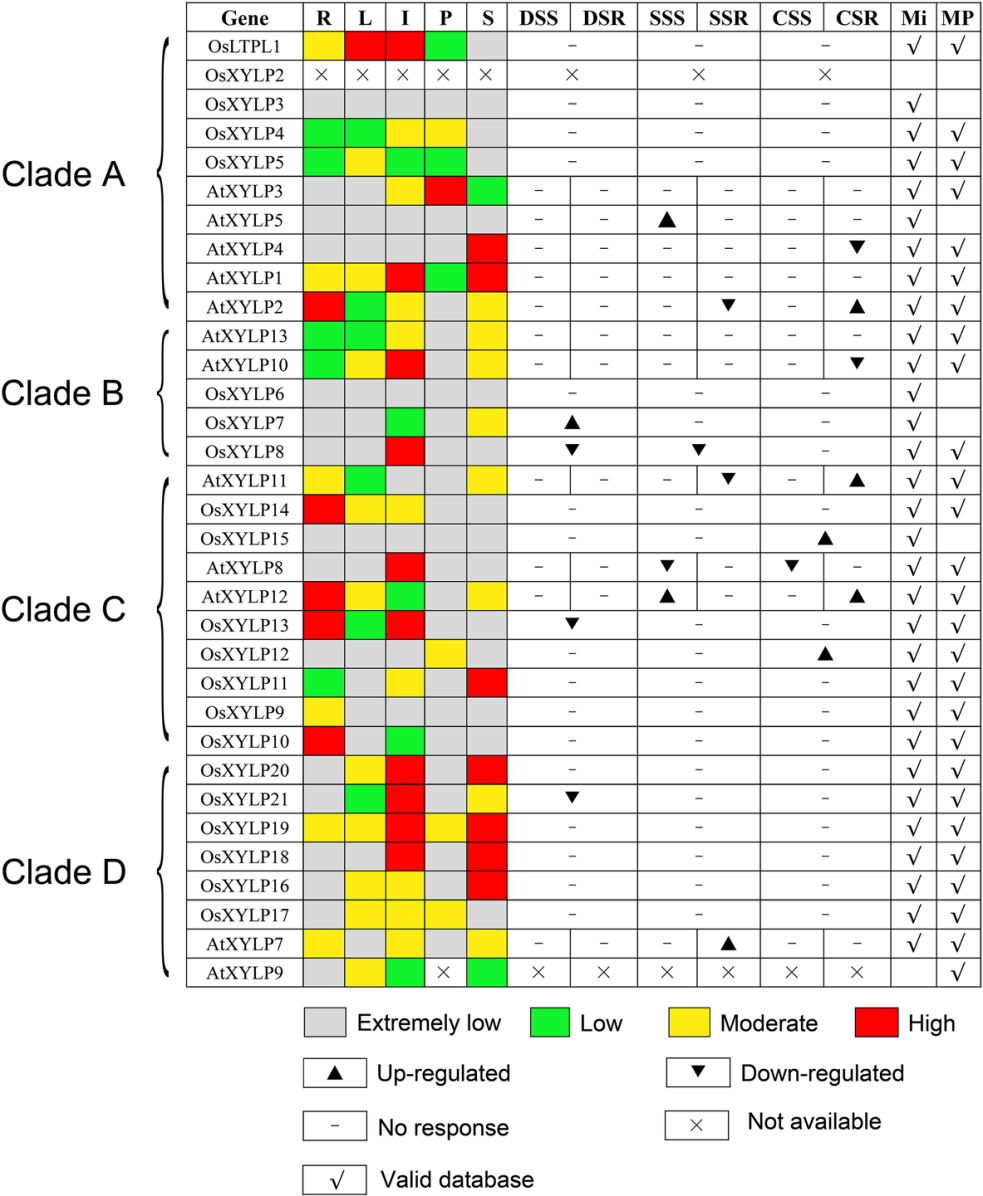
**Comparison of expression levels between rice and** ***Arabidopsis XYLP*** **genes in different organs and under abiotic stresses.** R, roots; L, leave; I, inflorescence; P, pollens; S, siliques or seeds; DSS and DSR; drought stressed shoots and roots; SSS and SSR, salt stressed shoots and roots; CSS and CSR, cold stressed shoots and roots; Mi, microarray data; MP, MPSS data.

To provide more evidence for the deduced biological functions of *XYLP* genes, a comparative expression analysis of rice and *Arabidopsis XYLP* genes was performed using microarray and MPSS data from roots, leaves, inflorescences, pollen, and siliques/seeds and from plants under abiotic stresses (Figure 8; Additional file 4: Table S3; Additional file 5: Table S4). All *OsXYLP* and *AtXYLP* genes were found to be present in at least one of the databases, except for *OsXYLP2* which was absent from the two data sets (Figure 8). Analysis of the integrated microarray and MPSS data revealed that 20 *XYLP* genes are expressed in at least two organs and tissues. Among the 20 genes, 6 *XYLP* genes showed specific expression patterns and 3 were entirely lowly expressed (Figure 8).

The analysis furthermore revealed that some *XYLP* genes with close evolutionary relationships have similar expression patterns. For example, *OsXYLP10*, *OsXYLP13*, *OsXYLP14*, and *AtXYLP12* are highly expressed in roots, as are *OsXYLP18*, *OsXYLP19*, and *OsXYLP20* in inflorescences and seeds (Figure 8).

It is noteworthy that *XYLP* genes originating from gene duplication events, such as, segmental duplicated genes: *OsXYLP6*, *OsXYLP7*, and *OsXYLP8*; *OsLTPL1* and *OsXYLP4*; tandem duplicated genes: *OsXYLP11* and *OsXYLP13*, *OsXYLP12* and *OsXYLP14*, do not show similar expression patterns and responses under abiotic stresses (Figure 8). These results are in accord with the conclusions of previous studies that the duplicated genes have frequently diverged from their ancestors, thus hinting that gene duplication has played an important evolutionary role by enriching biological functions of the *XYLP* gene family.

### Identification of *xylp7* and *xylp16* mutants

To investigate the biological functions of *OsXYLP* genes in rice, we acquired four T-DNA insertion mutants from the Plant Functional Genomics Laboratory of Korea. Two mutants (*xylp7* and *xylp16*) were successfully identified, and the expressions of *OsXYLP7* and *OsXYLP16* genes in their homozygous mutants were accordingly analyzed (Additional file 6: Figure S2).

**Figure 9:**
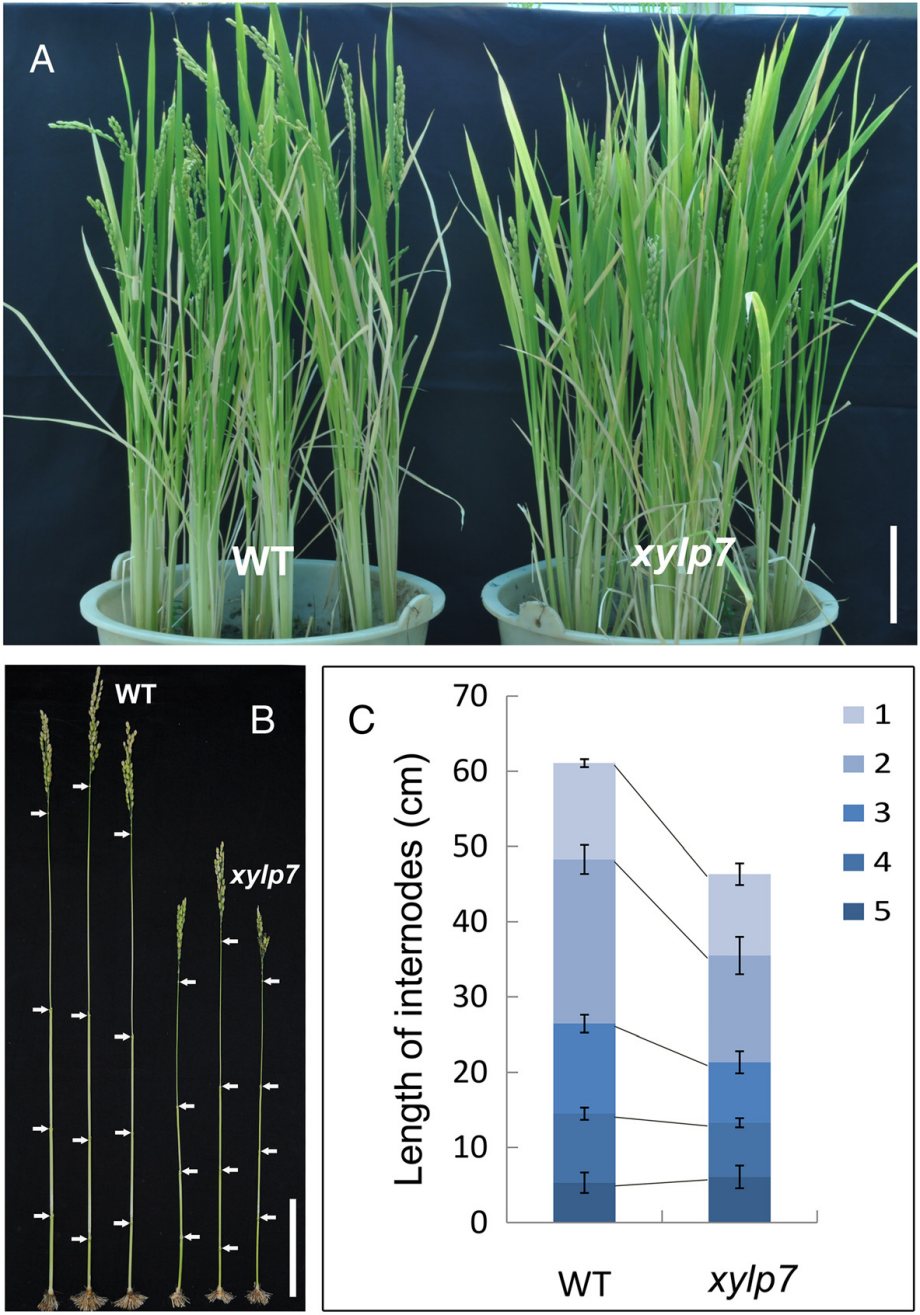
**Phenotypes of wild type and** ***xylp7*** **mutant plants. (A)** Plants at the mature stage. Scale bar: 10 cm. **(B)** The stem of the wild-type and *xylp7*. Scale bar: 10 cm. **(C)** Comparison of the internode lengths between the wild-type and mutant *xylp7.* Error bars indicate standard deviations of independent biological replicates (n =5 or more).

We observed and measured stem and spike stalk lengths of mature *xylp7* mutant plants. These lengths were found to be shorter in mutants than in the wild type, whereas no obvious distinction was observed in plant height (Figure 9A and B). The mutant *xylp7* plants displayed a reduction in the length of internodes, except for the basal internode (Figure 9C). We examined the expression level of *OsXYLP7* in different-aged stems by qRT-PCR. The results showed that *OsXYLP7* is high expressed in 70–90 day old stems and low expressed in 60-day-old stems (Additional file 7: Figure S3). The *xylp16* mutant plants showed no distinct phenotype compared with the wild type (data not shown).

## Discussion

In this study, we used ZeXYP1, AtXYP1, and AtXYP2 protein sequences to search for xylogen-like proteins in the RGAP database (http://rice.plantbiology.msu.edu/). After confirming the presence of nsLTP and AGP domains, we identified 21 *XYLP* genes in rice. The XYLP proteins were found to have a unique structure: chimeric AGPs with a conserved nsLTP domain. We classified *OsXYLP* genes into four clades based on their phylogenetic relationships, arranged their genetic information, and inferred their expression patterns from three conventional and valid bioinformatic databases. Observations of *xylp* mutants hinted that rice *XYLP* genes may have a function in the development of organs with vascular systems.

Gene duplication, both tandem and segmental, plays important roles in genome evolution [49]. *OsXYLP* genes are located on seven rice chromosomes. Thirteen (61.90%) of the 21 *OsXYLP* genes are derived from gene duplications: 9 genes attributed to segmental duplication are localized on chromosomes 1, 3, 5, and 7; and 3 pairs of tandemly duplicated genes are distributed on chromosomes 3 and 7 (Figure 3). Most duplicated *OsXYLP* genes show diverse expression patterns, including those arising from tandem and segmental duplications, such as tandemly duplicated gene pairs: *OsXYLP11* and *OsXYLP13*, *OsXYLP12* and *OsXYLP14*; and segmentally duplicated gene pairs: *OsXYLP6* and *OsXYLP8*, *OsXYLP9* and *OsXYLP17*. Our analyses indicate that the duplication events not only contributed to the expansion of the *OsXYLP* gene family, but also created differences in expression between duplicated genes that may have given rise to genetic functional diversity over the course of evolution.

Analysis of EST, microarray, and MPSS signature data revealed that all *OsXYLP* genes are expressed (Table 1). The analysis also indicated that most *OsXYLP* genes have high expression levels in tissues with vascular system, such as roots, stems, leaves, and panicles. OsXYLP and AtXYLP protein sequences were aligned and divided into four clades, with OsLTPL1, OsXYLP2, 3, 4, and 5 sharing high sequence homology with AtXYP1 and AtXYP2. A double mutant of *xyp1* and *xyp2* in *Arabidopsis*, but neither single mutant, shows defects in vascular development, and AtXYP1 has been confirmed to have TE-inducing activity [11]. *OsLTPL1* and *OsXYLP5* are highly expressed in roots, panicles, and seeds, similar to *AtXYP1*, and *OsXYLP4* and *AtXYP2* have similar expression patterns in roots (Figure 5). Thus, it is possible that *OsLTPL1*, *OsXYLP4*, and *OsXYLP5* function in the vascular system development of higher plants.

In previous studies, gibberellin plays roles in the control of cambial activity, differentiation of xylem fibers, and cell elongation of secondary xylem fibers [50]. Auxin and cytokinin coordinately post-transcriptionally regulate the accumulation of xylogen and are subsequently involved in the process of TE differentiation [11]. The hormones GA, auxin, and cytokinin have also been verified to affect secondary xylem development [51]. Studies indicate that hormones play important roles during plant vascular development [52]. In barley (*Hordeum vulgare* L.) aleurone protoplasts, β-GlcY inhibits GA-promoted induction of α-amylase, suggesting that AGPs are involved in GA function [53]. In our qRT-PCR analysis, all examined genes were up-regulated under GA treatment except for *OsXYLP5*, which showed little change. These results suggest that *OsXYLP* genes may participate in the hormone signaling pathway. Various genes have been reported to be involved in the GA signaling pathway and to have important roles in plant growth and development. For example, a rice mutant of the *Dwarf1* gene has dark green leaves, compact panicles, and short, round grains [54], and a gibberellin-responsive gene, *CsAGP1*, is involved in stem elongation [29]. In our study, we identified a *xylp7* mutant that has a significantly decreased stem height compared with the wild type, with every internode except for the basal internode observed to be shorter. These results suggest that *OsXYLP7* may take part in the GA signaling pathway and is likely to have an important role in stem elongation.

## Conclusions

We identified 21 *XYLP* genes from the rice genome and classified them into four clades according to their evolutionary relationships. We also elucidated their genomic characteristics, protein structures, duplication status, and expression patterns during different developmental stages as well as under abiotic stress treatments. Alterations in *OsXYLP* gene expression levels were observed under NAA, 6-BA, and GA treatments, indicating that *OsXYLP* genes may be involved in hormonal regulation. These data provide insights into the characteristics of *OsXYLP* genes. A mutant of *OsXYLP7* showed defects in stem length, suggesting that *OsXYLP7* has a function in the development of organs with vascular systems. In conclusion, this study has provided fundamental information on *OsXYLP* gene functions and is a first step in functional research of rice *XYLPs*. To our knowledge, this is the first report of xylogen-like proteins in *Oryza sativa* L., and 19 of the 21 identified *OsXYLPs* are new AGP genes.

## Methods

### Plant materials and treatment methods

*Oryza sativa* L. *japonica* cv. *Nipponbare* plants were cultivated in greenhouse at Wuhan University at 28°C with a 16 h light and 8 h dark cycle. Tissues and organs for expression analysis were: (i) 7-day-old roots (R, young root) and leaves (L, young leaves); (ii) 60-day-old stems (St, young stems); (iii) 5–10 cm panicles (P3) and (iv) 22–30 cm panicles (P6). For hormone treatments, the 7-day-old seedlings were transferred into deionized water contained 1 μM NAA (1-naphthylacetic acid), 5 μM 6-BA (6-Benzylaminopurine) or 5 μM GA (Gibberellin A_3_) for 3 h. For stress treatments, the 7-day-old seedling were transferred onto filter papers at 28°C as drought stress, placed in 400 mM NaCl solution at 28°C as salt stress, or stayed in sterile water at 4°C as cold stress for 3 h. The parallel control samples were kept the seedlings in sterile water at 28°C for 3 h. Stems of WT and *xylp7* mutant were collected respectively, including stems of 60, 70, 80, and 90 days-old plants. All materials above were respectively collected and frozen immediately in liquid nitrogen, and stored in −80°C until RNA extraction.

### Identification of OsXYLPs and bioinformatics analysis

Using the protein sequences of ZeXYP1, AtXYP1 and AtXYP2, BLAST searches (E-value < 10^−7^) were adopted to identify the OsXYLPs at the Rice Genome Annotation Project database (http://rice.plantbiology.msu.edu/). The results of three searches were integrated and then the redundant sequences were removed. The remaining protein sequences were submitted to InterProScan (http://www.ebi.ac.uk/Tools/InterProScan/) to make sure the presence of non-specific lipid transfer protein-like (nsLTP) domains. The presence of N-terminal signal peptide, GPI-anchored signal, and N-glycosylation sites were predicted on SignalP 3.0 (http://www.cbs.dtu.dk/services/SignalP/), Big-PI Plant Predictor (http://mendel.imp.ac.at/gpi/plant_server.html), and NetNGlyc 1.0 Server (http://www.cbs.dtu.dk/services/NetNGlyc/). Putative AG glycomodules were predicted mainly followed the criterion described in the papers [9,14,16]. Then, the protein sequences of identified OsXYLPs were used for BLASTP to ensure that all *XYLP* genes in available databases are identified. The characteristics of OsXYLP sequences were listed in Additional file 2: Table S1.

### Sequence and phylogenetic analysis

The sequences of non-specific lipid transfer protein-like (nsLTP) domains and full-length of OsXYLPs and AtXYLPs were aligned using DNAMAN and Clustal X (version 1.83) program, respectively. An un-rooted phylogenetic tree was generated in Clustal X using neighbor-joining method, the bootstrap value was 1000.

### Chromosomal localization and gene duplications

The approximate locations of *OsXYLP* genes were marking on the skeleton maps of rice chromosomes using the Mapchart software. Tandem duplicates genes were considered to be separated by no more than five genes. Genes belong to segmental duplicates were obtained from the “Segmental genome duplication of rice” at RGAP database (http://chibba.agtec.uga.edu/duplication/).

### Digital expression analysis

The EST expression data of *OsXYLP* genes were acquired from the UniGene database at NCBI (http://www.ncbi.nlm.nih.gov/unigene/). Genes whose EST number of any tissue occupied more than a half of the total values were deemed to specifically express.

The microarray data of *OsXYLPs* were obtained from the Rice Functional Genomic Express Database (http://signal.salk.edu/cgi-bin/RiceGE). Several tissues were chose for temporal and spatial analysis (GSE6893): YR, young roots; ML, mature leaf; YL, young leaf; SAM, shoot apical meristem; P1, 0–3 cm panicle, floral transition and floral organ development; P2 and P3, 3–5 cm and 5–10 cm panicle, meiotic stage; P4, 10–15 cm panicle, young microspore stage; P5, 15–22 cm panicle, vacuolated pollen stage; P6, 22–30 cm panicle, mature pollen stage; S1, 0–2 DAP (days after pollination) seed, early globular embryo; S2, 3–4 DAP seed, middle and late globular embryo; S3, 5–10 DAP seed, embryo morphogenesis; S4, 11–20 DAP seed, embryo maturation; S5, 21–29 DAP seed, dormancy and desiccation tolerance [48,55]. For abiotic stress analysis, rice seedlings were transferred to 200 mM NaCl solution for salt stress, dried on filter paper for drought stress, and stayed at 4°C for cold stress, for 3 h treatment respectively. The expression data of *AtXYLPs* were obtained from “Bulk Gene Download” at Nottingham *Arabidopsis* Stock Centre (http://www.ncbi.nlm.nih.gov/geo/info/download.html). Several tissues compared to those used in rice were selected: developmental stages (GSE5629-5633) and abiotic stresses treatments (GSE5620-5621 and 5623–5624). The absolute value of a gene in one tissue is divided by the average of all absolute values of all genes, and then the logarithms of the ratios from above procedure were used as input for cluster display take advantage of the Cluster and Treeview software.

The MPSS (massively parallel signature sequencing) data of *OsXYLP* and *AtXYLP* genes were obtained from the MPSS project (http://mpss.udel.edu). MPSS expression data representing different organs and tissues (9 in rice and 5 in *Arabidosis*) were used further analysis. The description of rice organs and tissues is: NYR, 14 day young roots; NRA, 60 day mature roots; NST, 60 day stems; NYL, 14 day young leaves; NME, 60 day meristem tissue; NPO, mature pollens; NOS, ovaries and mature stigmas; NIP, 90 day immature panicles; NCA, 35 day callus. The organs and tissues of *Arabidopsis* are: Ca, actively growing callus; In, inflorescence, mixed stages; L, 21 day leave; R, 21 day roots; Si, 24–48 hour post-fertilization siliques.

### Real-time PCR analysis

To confirm the expression of *OsXYLP* genes in rice tissues at different developmental stages and stress treatments identified by digital data analysis, quantitative real-time PCR (qRT-PCR) was performed by using SYBR-green fluorescence under a Rotor-Gene Q machine (Qiagen). The primer sequences are listed in Additional file 8: Table S5. The expression of genes in different samples was normalized to the expression of *UBQ5* housekeeping gene [56]. The relative expression levels were calculated using the standard curve method, a stand curve for each gene was built by using three times of 1, 3, 9, and 27 (from low to high) diluted series of a mixed cDNA pools [57]. At least two independent biological samples and three technical replicates of each biological sample were used for real-time PCR analysis.

### Availability of supporting data

Here we are with the supporting data (including alignments and protein sequences) as additional files. The phylogenetic data (alignments, phylogenetic trees, and protein structures) were deposited in Dryad (http://datadryad.org/). DOI: doi:10.5061/dryad.44tj3.

## Additional files

Additional file 1: Figure S1 Multiple sequence alignments of the nsLTP domain of OsXYLPs and AtXYLPs. Identical (100%), conservative (75-99%) and block (50-74%) of similar amino acid residues are shaded in black, red and light blue, respectively. Additional file 2: Table S1 Protein backbones of XYLPs in rice and *Arabidopsis*. Additional file 3: Table S2 ESTs expression profiles of *OsXYLP* genes. Additional file 4: Table S3 MPSS analysis of *OsXYLP* and *AtXYLP* genes. Additional file 5: Table S4 Microarray analysis of *OsXYLP* and *AtXYLP* genes. Additional file 6: Figure S2 Analyses of T-DNA insertion in the *OsXYLP7* and *OsXYLP16* genes. (A) Genomic drawing of *OsXYLP7* locus and the position of T-DNA insertion. The T-DNA insertion of *xylp7* allele represented in triangle inserted in the second exon (E2). Bar = 500 bp. (B) Genomic drawing of *OsXYLP16* locus and the position of T-DNA insertion. The T-DNA insertion of *xylp16* allele represented in triangle inserted in the first exon (E1). Bar = 500 bp. (C) and (E) PCR analysis of T-DNA insert in *xylp7* and *xylp16* mutant, respectively; wild type (WT) is as the control test. (D) and (F) RT-PCR analysis of expression level in *xylp7* and *xylp16* mutant, respectively; wild type (WT) is as the control test. Additional file 7: Figure S3 The expression levels of OsXYLP7 in different stages of stems. Additional file 8: Table S5 Primers used in real-time PCR and mutants identification.

## Abbreviations

AGPs: Arabinogalactan proteins
AG: Arabinogalactan
FLAs: Fasciclin-like AGPs
TE: Tracheary elements
RAP-DB: Rice annotation project database
MPSS: Massively parallel signature sequencing
EST: Expressed sequence tag
NAA: 1-naphthylacetic acid
6-BA: 6-Benzylaminopurine
GA: Gibberellin A_3_
β-GlcY: β-glucosyl Yariv reagent
DAP: Day after pollination
