# Supplementary figures and images for: Identification, characterization, and transcription analysis of xylogen-like arabinogalactan proteins in rice (Oryza sativa L.)
Source: BMC Plant Biol. 2014 Nov 18;14:299. doi: 10.1186/s12870-014-0299-y (PMC4239379; doi:10.1186/s12870-014-0299-y)

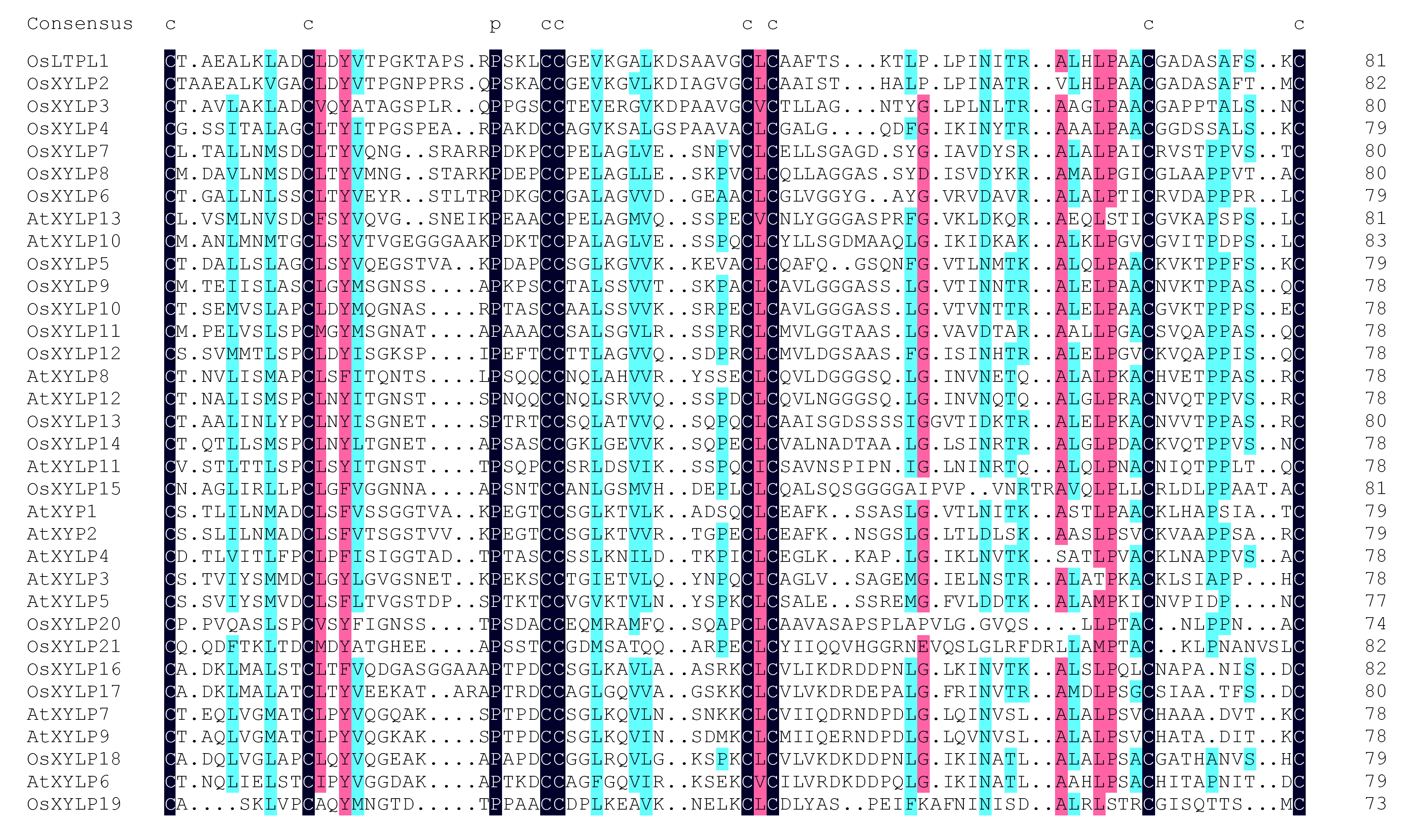

Supplement: Additional file 1: Figure S1 — Multiple sequence alignments of the nsLTP domain of OsXYLPs and AtXYLPs. Identical (100%), conservative (75-99%) and block (50-74%) of similar amino acid residues are shaded in black, red and light blue, respectively. [file 12870_2014_299_MOESM1_ESM.jpeg]

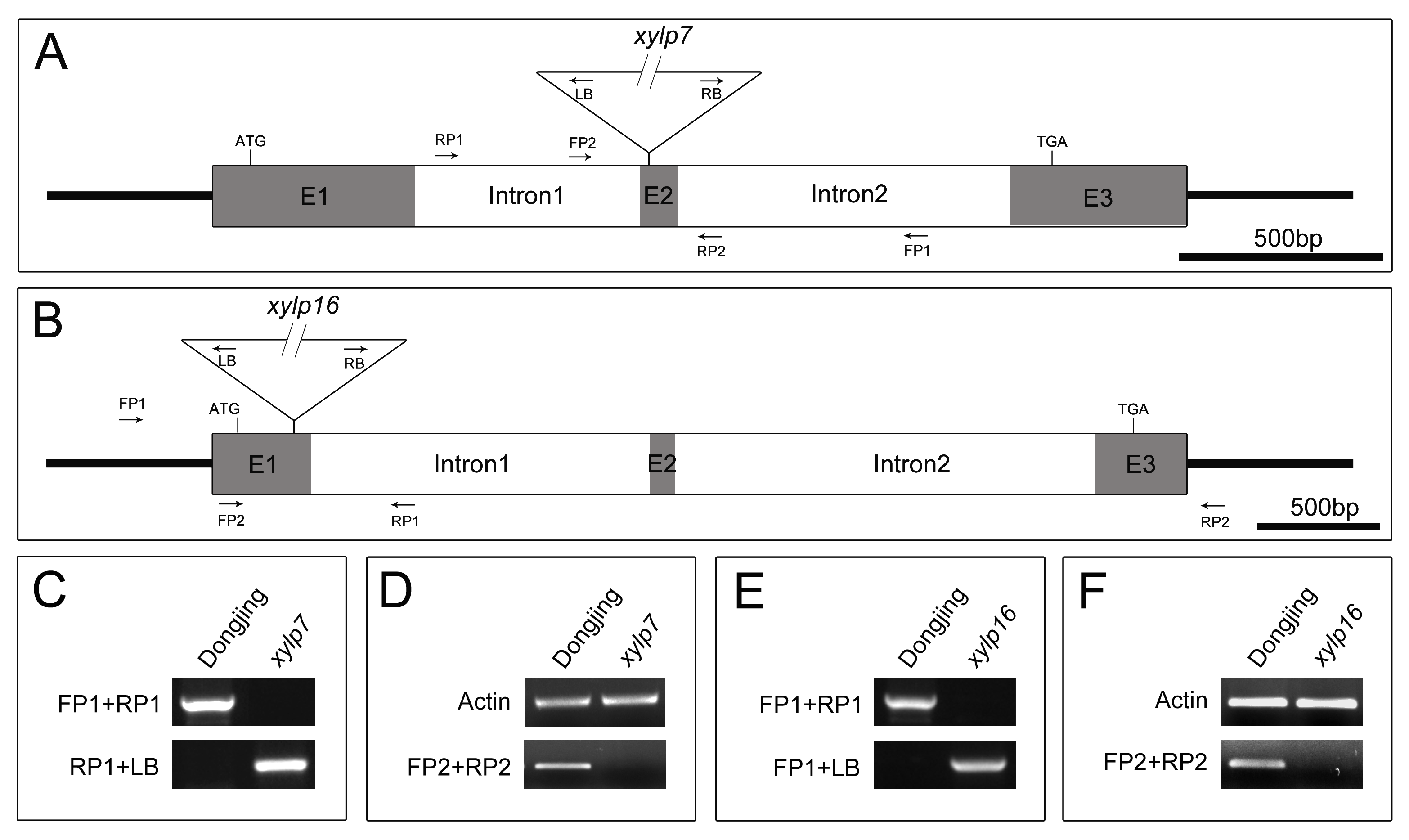

Supplement: Additional file 6: Figure S2 — Analyses of T-DNA insertion in the OsXYLP7 and OsXYLP16 genes. (A) Genomic drawing of OsXYLP7 locus and the position of T-DNA insertion. The T-DNA insertion of xylp7 allele represented in triangle inserted in the second exon (E2). Bar = 500 bp. (B) Genomic drawing of OsXYLP16 locus and the position of T-DNA insertion. The T-DNA insertion of xylp16 allele represented in triangle inserted in the first exon (E1). Bar = 500 bp. (C) and (E) PCR analysis of T-DNA insert in xylp7 and xylp16 mutant, respectively; wild type (WT) is as the control test. (D) and (F) RT-PCR analysis of expression level in xylp7 and xylp16 mutant, respectively; wild type (WT) is as the control test. [file 12870_2014_299_MOESM6_ESM.tiff]

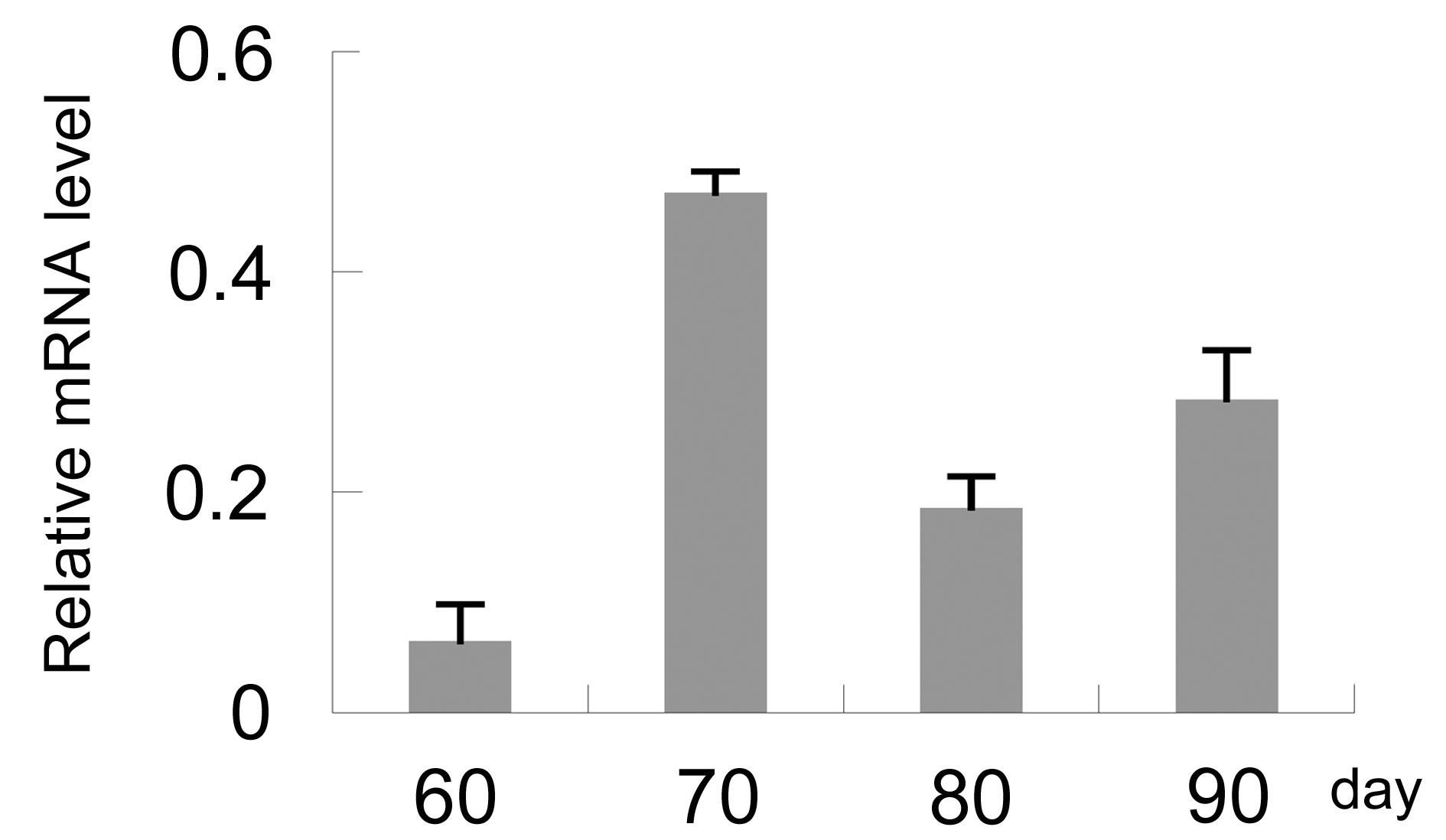

Supplement: Additional file 7: Figure S3 — The expression levels of OsXYLP7 in different stages of stems. [file 12870_2014_299_MOESM7_ESM.jpeg]
