# Supplementary material for: Identification, characterization, and transcription analysis of xylogen-like arabinogalactan proteins in rice (Oryza sativa L.)
Source: BMC Plant Biol. 2014 Nov 18;14:299. doi: 10.1186/s12870-014-0299-y (PMC4239379; doi:10.1186/s12870-014-0299-y)
Supplement: Additional file 2: Table S1 — Protein backbones of XYLPs in rice and Arabidopsis. [file 12870_2014_299_MOESM2_ESM.doc]

**Table S1. Protein backbones of XYLPs in rice and *Arabidopsis***

| Name | Locus | Protein backbones |
| --- | --- | --- |
| OsLTPL1 | LOC_Os03g26820 | MAVAARAAAVACLLVVGLAAVAGVDGATASSPAPAPAVD**CTAEALKLADCLDYVTPGKTAPSRPSKLCCGEVKGALKDSAAVGCLCAAFTSKTLPLPINITRALHLPAACGADASAFSKC**LAPAPSPSVAPGTSSGSGGAAAAPAKGAAAARSPMASTTAVLVVAAAVAAPLLAFFHF |
| OsXYLP2 | LOC_Os03g26800 | MAVAAGSAAVACLLVVLGLAAVAGVDGATASSHPAPAPAPAPAVD**CTAAEALKVGACLDYVTPGNPPRSQPSKACCGEVKGVLKDIAGVGCLCAAISTHALPLPINATRVLHLPAACGADASAFTMC**LGQSTYFDLLLL |
| OsXYLP3 | LOC_Os07g30590 | MQRGAATAMPTLLMTIFLVALVSGGRVASQPQPQEAPAPAPEGTGSSSGA**CTAVLAKLADCVQYATAGSPLRQPPGSCCTEVERGVKDPAAVGCVCTLLAGNTYGLPLNLTRAAGLPAACGAPPTALSNC**NVPSPKGGDRSGSSPKSTATPAPITIVVFVATVVAVFCYL |
| OsXYLP4 | LOC_Os07g43290 | MSRAVVVVVVVAVLALACGAASQSPAPAPAAGPASD**CGSSITALAGCLTYITPGSPEARPAKDCCAGVKSALGSPAAVACLCGALGQDFGIKINYTRAAALPAACGGDSSALSKC**NKKFPGASPTGAPAPSSSGSGSGSTPATGTPSSPKSAAAQSPVSAMLIVATVAAPLLSYYYL |
| OsXYLP5 | LOC_Os03g09230 | MAGAGRTRSTAMAAWSYSSLLLQLLLLSMVAVLDGAATTGGGGAGAPAPAAD**CTDALLSLAGCLSYVQEGSTVAKPDAPCCSGLKGVVKKEVACLCQAFQGSQNFGVTLNMTKALQLPAACKVKTPPFSKC**HLSIPGVTGGAPAPAPFSGAPFFGGSSPSASPAGTGSDSAAATVRAPAPSPSAAVRPKETKAALFSAAVIAAATLLAHRA |
| OsXYLP6 | LOC_Os03g20760 | MAMIMLPAASAAAAASVLALLVLTSLQPRAARAQVASSPWAAPAPWPGELD**CTGALLNLSSCLTYVEYRSTLTRPDKGCCGALAGVVDGEAACLCGLVGGYGAYGVRVDAVRALALPTICRVDAPPPRLC**AALGVPVAEPPGGAVPEESGLSGGMPANAPSTAATGSSGGGGPATHRPTRRHLILLLLLLVFPASLLLL |
| OsXYLP7 | LOC_Os05g41030 | MAAQRRWPTSTLAAVAVAVVVLLAASAATTAEAQSAPAAAPGPAGPVLDQA**CLTALLNMSDCLTYVQNGSRARRPDKPCCPELAGLVESNPVCLCELLSGAGDSYGIAVDYSRALALPAICRVSTPPVSTC**AAFGFNVPMGPTPSPSPAAVSPSGEGPQFPGTSPFASPPSTATPSTNAAAAGRSGDHLVAVGVAIAAAAVVVAGMFRIV |
| OsXYLP8 | LOC_Os01g59870 | MALTVRLHTAVAAVAVAVVVAMGMAAAQMSPAGAPAPAGGISPA**CMDAVLNMSDCLTYVMNGSTARKPDEPCCPELAGLLESKPVCLCQLLAGGASSYDISVDYKRAMALPGICGLAAPPVTAC**ALLGVPVPMAPSASPMAGLGPSTEPQMPEKSPSASPSESSNHAPGRFTALAAVVLAVAAAGMV |
| OsXYLP9 | LOC_Os07g07790 | MAVARGVALAVVLAAAAAILAASPVAAQGGGGGGGSGS**CMTEIISLASCLGYMSGNSSAPKPSCCTALSSVVTSKPACLCAVLGGGASSLGVTINNTRALELPAACNVKTPPASQC**STVGVPMPSPATPATPAAPAVPSETPAGTGGSKATPTTATTTTGQSASGGSVGKAASMATVVVSVAFALIHV |
| OsXYLP10 | LOC_Os07g07860 | MARNNGVAVMFAAVVVVAGALVAGAAAQSG**CTSEMVSLAPCLDYMQGNASRPTASCCAALSSVVKSRPECLCAVLGGGASSLGVTVNTTRALELPAACGVKTPPPSEC**SKVGAPIPSPAPGGAAAPNAPPAAGTGSKTTPTTGASSAGESVGKAASVAMVIVSAAFAMLYA |
| OsXYLP11 | LOC_Os03g57990 | MGAGRGSNGAVLGIGVAVVTALLAWRCAAAAAQAPPVASTDGGGSG**CMPELVSLSPCMGYMSGNATAPAAACCSALSGVLRSSPRCLCMVLGGTAASLGVAVDTARAALLPGACSVQAPPASQC**NAAGVPVSSPA**N**PTTSGGTPATPAGTPGSKTTPASTTQYSDGSVNRSRVILVILVAAIVVFLDHF |
| OsXYLP12 | LOC_Os07g07870 | MAARKSQTGVPRAPVVAVIVVVMTMLASRAASQNNG**CSSVMMTLSPCLDYISGKSPIPEFTCCTTLAGVVQSDPRCLCMVLDGSAASFGISINHTRALELPGVCKVQAPPISQC**TAVPTPPPAPDTPTLADEPAETNEDEPSPPPAGSAGS**N**KTSSATNSKKAASLMASVLIPTCALFYVF |
| OsXYLP13 | LOC_Os03g57970 | MAAAGVSGLAVGCLVAATAALLVAGASAQTG**CTAALINLYPCLNYISGNETSPTRTCCSQLATVVQSQPQCLCAAISGDSSSSIGGVTIDKTRALELPKACNVVTPPASRC**NSAGGNTPGAATTTSPATQTPGATGAGTGVGSKTTPTAPYLINGGASLRGATGLVLALAAVAVYAV |
| OsXYLP14 | LOC_Os07g07930 | MAARVAWIGAAAAVVVVALMAGGAAAQPPSSTSG**CTQTLLSMSPCLNYLTGNETAPSASCCGKLGEVVKSQPECLCVALNADTAALGLSINRTRALGLPDACKVQTPPVSNC**KSGAAAPPAGQTPTTPAGTGSKATPATPVGSGVAPLRVSPVGILAGIVVAAVYAVSAV |
| OsXYLP15 | LOC_Os04g38840 | MASSAVVAACVVVVAAALLLVTAPGAAAQPGGASSGSG**CNAGLIRLLPCLGFVGGNNAAPSNTCCANLGSMVHDEPLCLCQALSQSGGGGAIPVPVNRTRAVQLPLLCRLDLPPAATAC**PGFDLGGAAPSPPVSVPRSTPNSTAPSTPTPVTVTRAPPQQMTPSPKTSSQTPEYSSGLKLIADCVPVALGFMALVSALTF |
| OsXYLP16 | LOC_Os07g09970 | MEHHRHRGAVVLVVAAAAMAMAMSAVRGDFAADRAE**CADKLMALSTCLTFVQDGASGGAAAPTPDCCSGLKAVLAASRKCLCVLIKDRDDPNLGLKINVTKALSLPQLCNAPANISDC**PRLLNLPPNSKDAQIFEQFAKQQAAMQGSPSASPGGSSAPAAGAQKSGAAVLRWLGVDGVGGGGARAVALLLFLLSSAVAVAAPLLLVF |
| OsXYLP17 | LOC_Os03g58940 | MAAAARWWVAAVVVAVAAMAGAAKGDFAADKAE**CADKLMALATCLTYVEEKATARAPTRDCCAGLGQVVAGSKKCLCVLVKDRDEPALGFRINVTRAMDLPSGCSIAATFSDC**PKMLNMSPDSKEAEIFKQYAREHESN**N**ATKPAPAAAAAATGSAGKATAATGDAGVGRRQRSSLAARAVAAAVLAAVFGLTVA |
| OsXYLP18 | LOC_Os03g07100 | MAMVAMVVAAMAVAAVARGDMSADRTE**CADQLVGLAPCLQYVQGEAKAPAPDCCGGLRQVLGKSPKCLCVLVKDKDDPNLGIKINATLALALPSACGATHANVSHC**PQLLHIPPNSKDAAIFSPGGDKGSPAAPAKD**N**STTTTDSRAVQAANGGSRSSAATAGAALTALLAGYFLLLLPEFSAPSSF |
| OsXYLP19 | LOC_Os06g47200 | MATRMAAAVAAMVAAVAISLAAGGAAQSSPSTPS**CASKLVPCAQYMNGTDTPPAACCDPLKEAVKNELKCLCDLYASPEIFKAFNINISDALRLSTRCGISQTTSMC**PGNSPTNSPPASPSGGKNAGHRTMSVGLPGLMSLFLALWSVLA |
| OsXYLP20 | LOC_Os03g46150 | MDRSMARRRRGTSFTASWQLGLAVVVAAIMASSAQPQQQQQQPPQPPGQPANAPS**CPPVQASLSPCVSYFIGNSSTPSDACCEQMRAMFQSQAPCLCAAVASAPSPLAPVLGGVQSLLPTACNLPPNAC**AGKPFVRAVPILTCGLNSRTHGVIAVAGVVFADATGSTSGSAPAGGSSATPSTGATAAAPAMEPAGMDPAMTAGGGSKSVPGMPYSAAAGVHGGGASAAVAVLISSMLAYACMI |
| OsXYLP21 | LOC_Os08g42040 | MAAWRGLALAAVVAWCVAAAAAAPDAALQSK**CQQDFTKLTDCMDYATGHEEAPSSTCCGDMSATQQARPECLCYIIQQVHGGRNEVQSLGLRFDRLLAMPTACKLPNANVSLC**INLLHLTPSSPDYAVFANASKAAATTPSSTTPGAAAATAGGFKVQAGLSYGVVAAAMVSAVFSSIF |
| AtXYP1 | At5g64080 | MATHSSFTATTPLFLIVLLSLSSVSVLGASHHHATAPAPSVD**CSTLILNMADCLSFVSSGGTVAKPEGTCCSGLKTVLKADSQCLCEAFKSSASLGVTLNITKASTLPAACKLHAPSIATC**GLSVAPSTAPGLAPGVAAAGPETAGFLAP**N**PSSGNDGSSLIPTSFTTVLSAVLFVLFFSSA |
| AtXYP2 | At2g13820 | MAYATILMIFSVVALMSGERAHAAVD**CSSLILNMADCLSFVTSGSTVVKPEGTCCSGLKTVVRTGPECLCEAFKNSGSLGLTLDLSKAASLPSVCKVAAPPSARC**GLSVSGDPPATAPGLSPTAGAGAPALSSGANAATPVSSPRSSDASLLSVSFAFVIFMALISSFY |
| AtXYLP3 | At4g08670 | MKQSLLLSFVLLLLSSSSLVTPIHAR**N**KSNPAKSPVGAPAPGPSSSD**CSTVIYSMMDCLGYLGVGSNETKPEKSCCTGIETVLQYNPQCICAGLVSAGEMGIELNSTRALATPKACKLSIAPPHC**GIITSGATTPGASPVSPSAGAPTTSPSAAKSPETSATSPSSDETPSMTAPSPSSSGTNILSVPALTIVFVIVSSVAYISAFSN |
| AtXYLP4 | At5g09370 | MAYFSTATSLLLLVLSVSSPYVHGASD**CDTLVITLFPCLPFISIGGTADTPTASCCSSLKNILDTKPICLCEGLKKAPLGIKLNVTKSATLPVACKLNAPPVSAC**DSLPPASPPTANGQAPVWGSGWAPAPSPSKGNSLIPISGFSFVIVTALAMFRI |
| AtXYLP5 | At1g36150 | MKPSFVLLSIVLLLSSSLSDAADFGSPSQPPSMAPTPQPS**N**STD**CSSVIYSMVDCLSFLTVGSTDPSPTKTCCVGVKTVLNYSPKCLCSALESSREMGFVLDDTKALAMPKICNVPIDPNC**DVSTPAASTPVSPPVESPTTSPSSAKSPAITPSSPAVSHSPPPVRHSSPPVSHSSPPVSHSSPPTSRSSPAVSHSSPVVAASSPVKAVSSSTASSPRAASPSPSPSPSISSSGILLVSKLFIAVVMVSSFLYILA |
| AtXYLP6 | At1g55260 | MIPSSNQYVDTHRVTEDRAIYIHEHTKRLEDTFLSRENTTHRTMEKSTRTLFITIVITSMLLGFGNSDLAQDREE**CTNQLIELSTCIPYVGGDAKAPTKDCCAGFGQVIRKSEKCVCILVRDKDDPQLGIKINATLAAHLPSACHITAPNITDC**ISILHLPRNSTLAKEFENLGRIEDNYNSTSPTQIHKDGTGGGKAEPVKSNGWKEKSWLGVELLIYLLVSLIFF |
| AtXYLP7 | At2g44300 | MESRKINLMATAIALIVVAMVVAAADDKTKDKEE**CTEQLVGMATCLPYVQGQAKSPTPDCCSGLKQVLNSNKKCLCVIIQDRNDPDLGLQINVSLALALPSVCHAAADVTKC**PALLHLDPNSPDAQVFYQLAKGL**N**KTGPASAPTGSSPGPISISPTSGSDDGNNSGRTTSVPGRNHAQSFYKQWLGLEVVFHFFVIFYIFILV |
| AtXYLP8 | At4g14815 | MKPRMCLILFIALMRVMSIVSAQSS**CTNVLISMAPCLSFITQNTSLPSQQCCNQLAHVVRYSSECLCQVLDGGGSQLGINVNETQALALPKACHVETPPASRC**HSGSSVNSHSEHG**N**GSKTVPREKSSSDGSIKFSFPLLAILFTASYITLIYAKY |
| AtXYLP9 | At2g44290 | MESRKIKVMATAIALIMVAMVVDAAGADKGKDKEE**CTAQLVGMATCLPYVQGKAKSPTPDCCSGLKQVINSDMKCLCMIIQERNDPDLGLQVNVSLALALPSVCHATADITKC**PALLHLDPNSPDAQVFYQLAKGL**N**ETVSASAPTGSASEPTSMSSTPGSSAG**N**NSGRTTSVPGTNHAQSFSKQWLGLEVVAHFFVIFYIFILV |
| AtXYLP10 | At3g43720 | MSNVVVIAVVLIVASLTGHVSAQMDMSPSSGPSGAPD**CMANLMNMTGCLSYVTVGEGGGAAKPDKTCCPALAGLVESSPQCLCYLLSGDMAAQLGIKIDKAKALKLPGVCGVITPDPSLC**SLFGIPVGAPVAMGDEGASPAYAPGSMSGAESPGGFGSGPSASRGSDAPSSAPYSLFLNLIIFPLAFAFYIFC |
| AtXYLP11 | At2g48130 | MGYRRSYAITFVALVAALWSVTKAQPSSS**CVSTLTTLSPCLSYITGNSTTPSQPCCSRLDSVIKSSPQCICSAVNSPIPNIGLNINRTQALQLPNACNIQTPPLTQC**NAATGPTAQPPAPSPTEKTPDVTLTPTSLPGARSGVGGGSKTVPSVGTGSSSRNVDPLPLHFLMFAVLVVCTSSFL |
| AtXYLP12 | At3g22600 | MKMEMGLVFLTVFMAVMSSTMVSAQSS**CTNALISMSPCLNYITGNSTSPNQQCCNQLSRVVQSSPDCLCQVLNGGGSQLGINVNQTQALGLPRACNVQTPPVSRC**NTGGGGGGSTSDSPAESPNSSGPG**N**GSKTVPVGEGDGPPSSDGSSIKFSFPLIAFFSAVSYMAIF |
| AtXYLP13 | At2g27130 | MLTTNTLAVLLLLFLSLCSGQSPPAPEPIAADGPSSPVN**CLVSMLNVSDCFSYVQVGSNEIKPEAACCPELAGMVQSSPECVCNLYGGGASPRFGVKLDKQRAEQLSTICGVKAPSPSLC**SVLGFPTISPAGSEDSSSGSEGSDKDKKNGAMTTKYCGVALNSLALLLLFTFLSLS |

**Supplementary Figure S3**. Sequences in the boxes indicate the N-terminal signal peptide. Sequences colored in light blue indicate the 8-Cys motif. Sequences in black bold font and red bold font indicate the nsLTP domain and the N-glycosylation site, respectively. Sequences with black underline and red double underline indicate the putative AG site and arabinosylation site, respectively. Sequences in deep gray indicate the C-terminal GPI–anchor.
