# Supplementary material for: Identification, characterization, and transcription analysis of xylogen-like arabinogalactan proteins in rice (Oryza sativa L.)
Source: BMC Plant Biol. 2014 Nov 18;14:299. doi: 10.1186/s12870-014-0299-y (PMC4239379; doi:10.1186/s12870-014-0299-y)
Supplement: Additional file 8: Table S5 — Primers used in real-time PCR and mutants identification. [file 12870_2014_299_MOESM8_ESM.doc]

**Table S5. Primers used in real-time PCR and mutant identification**

| Primer name | Primer sequence (5′-3′) |
| --- | --- |
| *UBQ5*-FP | ACCACTTCGACCGCCACTACT |
| *UBQ5*-RP | ACCACTTCGACCGCCACTACT |
| *OsLTPL1*-FP | CAGCGAGGTCTCCGATGG |
| *OsLTPL1*-RP | GAATGTAATGAGGCTACGAAA |
| *OsXYLP4*-FP | CGCCCTGAGCAAGTGCAACAAG |
| *OsXYLP4*-RP | TGGCCGGTGTTGATCCTGAC |
| *OsXYLP5*-FP | CCGCCGCCACCGTAAGAGCT |
| *OsXYLP5*-RP | CGGCGATTACAGCGGCAGAGAA |
| *OsXYLP6*-FP | CGACGCACCGACCCACAAGAC |
| *OsXYLP6*-RP | TGTGCTGCTTGCCGGTGTGAC |
| *OsXYLP7*-FP | GTGGCTGTTTGTTTGTATGG |
| *OsXYLP7*-RP | GTCATGGAATCCAGCAAGT |
| *OsXYLP8*-FP | CCGACGATTCTTGTTTATTCT |
| *OsXYLP8*-RP | GCAACTTACGACAAGGAAGAGT |
| *OsXYLP9*-FP | GCCGCCTGCAACGTCAAGA |
| *OsXYLP9*-RP | CGCCGGAGTCTCGCTTGG |
| *OsXYLP10*-FP | CCCAGCGAGTGCAGCAAAGTC |
| *OsXYLP10*-RP | CGGTGGTCGGCGTCGTCTTC |
| *OsXYLP11*-FP | CGAACCCGACGACATCC |
| *OsXYLP11*-RP | AACGCTTCCGTCAGAGTATTG |
| *OsXYLP12*-FP | CGCCGATCCCCGAGTTCAC |
| *OsXYLP12*-RP | GCGCCCTCGTGTGGTTGATG |
| *OsXYLP13*-FP | AGCGTCTGTCTGCTACTCTGA |
| *OsXYLP13*-RP | TTGCCAATGCTCAATGATGC |
| *OsXYLP14*-FP | GCCTATCACCGTCTGTATCAA |
| *OsXYLP14*-RP | CATAAACCAATGGAGGAAGAA |
| *OsXYLP15*-FP | GCCGCCGCAACAGATGACAC |
| *OsXYLP15*-RP | CGGCACAATTCTAATCTCCCCTAA |
| *OsXYLP16*-FP | CCCGCCAACATCTCCGACTG |
| *OsXYLP16*-RP | TCCGTCCACTCCCAACCA |
| *OsXYLP17*-FP | TCCGTGCTGACGAGTGAC |
| *OsXYLP17*-RP | CAGCCTGGAAAGGAAAGC |
| *OsXYLP18*-FP | GGACAATGCCTGGTGAAA |
| *OsXYLP18*-RP | TCCAGAACATAGCAGGAAAA |
| *OsXYLP19*-FP | TTCCAGGATTGATGAGC |
| *OsXYLP19*-RP | TCTTCTGACTTGCGACA |
| *OsXYLP20*-FP | TGCCGTCGCCGTCTTGAT |
| *OsXYLP20*-RP | TGCATATACCATCACGATCACAA |
| *OsXYLP21*-FP | GGCGGCTTCAAGGTTCA |
| *OsXYLP21*-RP | GTGGGTGACTAGCTTGTTTCA |
| *xylp7*-FP1 | TTGCGTGTATAAGGCAAAGC |
| *xylp7*-RP1 | GCAGTAGCAGCAGCACTCTC |
| *xylp7*-FP2 | TTTGGTGAGGGTATTTGCTTTTGA |
| *xylp7*-RP2 | CCTGCCTTAGCCATCACTGACAA |
| *xylp16*-FP1 | CTTCAAAATTCATGGGTCGG |
| *xylp16*-RP1 | ATATATTGCGTGCCTGAGCC |
| *xylp16*-FP2 | CGGCGGACAGGGCGGAGT |
| *xylp16*-RP2 | CGGCGGAGGAGAGGAGGAAGAG |
